# Lessons from Canada’s notice of compliance with conditions policy for the life-cycle regulation of drugs

**DOI:** 10.1093/jlb/lsad008

**Published:** 2023-04-12

**Authors:** Melanie McPhail, Howard Zhang, Zohra Bhimani, Tania Bubela

**Affiliations:** Simon Fraser University, Faculty of Health Sciences, Burnaby, Canada; Simon Fraser University, Faculty of Health Sciences, Burnaby, Canada; Simon Fraser University, Faculty of Health Sciences, Burnaby, Canada; Simon Fraser University, Faculty of Health Sciences, Burnaby, Canada

**Keywords:** conditional regulation, drug regulation, health law, health technologies, life-cycle regulation, regulatory science

## Abstract

Innovative health technologies are not well regulated under current pathways, leading regulators to adopt contextual, life-cycle regulatory models, which authorize drugs based on earlier clinical evidence subject to the conduct of post-market trials that confirm clinical benefit and safety. In this paper, we evaluate all drugs authorized in Canada under the Notice of Compliance with conditions (NOC/c) policy from 1998 to 2021 to analyze its function, identify challenges and areas for improvement, and make recommendations to inform Health Canada’s regulatory reforms. We analyzed a sample of 148 drugs authorized between 1998 and 2021, including characteristics about the pre- and post-market clinical trials, finding that most NOC/c authorizations are based on one, single-arm clinical trial using a surrogate endpoint. Post-market trials are more likely to be randomized, Phase III trials but mostly use surrogate endpoints. Based on our findings, we recommend increasing decision-making transparency throughout the regulatory process, developing comprehensive eligibility criteria for selecting appropriate health technologies, modernizing pre-market evidence requirements, adopting a more active role in designing post-market trials, and utilizing automatic expiry, stronger penalties, and ongoing disclosure of the status of post-market trials to promote compliance.

## I. INTRODUCTION

Pharmaceutical and biotechnology industries, together with academic researchers, are developing innovative health technologies not well regulated under current pathways. In response, regulators are transitioning from rules-based, *ex-ante* regulatory authorization models to contextual, life-cycle regulatory models. Life-cycle regulatory pathways are those that authorize drugs based on earlier clinical evidence, subject to the conduct of post-market trials that confirm clinical benefit and safety. Drug regulators around the world have used life-cycle regulatory mechanisms, including conditional and expedited pathways, to authorize new drugs for decades, most commonly for new cancer drugs. Existing life-cycle regulatory pathways have been analyzed and critiqued, primarily in the United States and European Union.^1^ However, analysis of Canada’s Notice of Compliance with conditions (NOC/c) policy has been limited.^2^ In 2021, Health Canada announced its intent to substantially reform its regulatory frameworks for drugs and devices, including broader use of terms and conditions placed on drug and device authorizations. These regulatory amendments will replace the NOC/c policy and create a life-cycle regulatory framework. It is therefore timely to evaluate all drugs authorized under the NOC/c policy from 1998 to 2021 to analyze its function, identify challenges and areas for improvement, and make recommendations to inform Health Canada’s intended regulatory reforms.

## II. LIFE-CYCLE REGULATORY AUTHORIZATION IN CANADA

Health Canada introduced its NOC/c policy in May 1998, following pressure to align with the changing regulatory norms in the United States.^3^ The processes and rules of the NOC/c policy are set out in the *Notice of Compliance with Conditions (NOC/c) Guidance Document*, an administrative instrument that does not have the force of law. Guidance documents are designed ‘to provide assistance to industry and health care professionals on how to comply with the policies and governing statutes and regulations …[and] provide review and compliance guidance to staff, thereby ensuring that mandates are implemented in a fair, consistent and effective manner.’^4^ Currently, neither the Food and Drugs Act^5^ nor the Food and Drug Regulations^6^ references the NOC/c policy.

The objective of the NOC/c policy is to *‘provide access to promising new drugs for patients suffering from serious, life-threatening or severely debilitating disease or conditions for which no drug is presently marketed in Canada or for which a significant increase in efficacy or a significant decrease in risk is demonstrated in relation to an existing drug marketed in Canada.*’^7^ The two purported benefits of the NOC/c policy are: (1) to facilitate earlier access to drugs; and (2) to permit enhanced post-market surveillance initiatives to monitor and report on the safety and efficacy of promising new therapies. Authorization under the NOC/c policy is available for new active substances that have not been previously authorized, new indications for previously authorized therapeutic products, or generic versions of previously authorized therapeutic products.^8^

Eligibility for the NOC/c pathway is based on a disease criterion and a treatment landscape criterion. Under the disease criterion, Health Canada has discretion to determine whether a drug targets a serious or life-threatening disease or a severely debilitating disease. The policy specifically contemplates that HIV/AIDS, amyotrophic lateral sclerosis, and some cancers qualify as serious or life-threatening diseases, but does not exclude other diseases or conditions. The drug must also be indicated for the treatment, prevention, or diagnosis of serious symptoms or manifestations of the condition, rather than a minor irritation or symptoms resulting from the condition. Severely debilitating disease may include chronic conditions such as inflammatory bowel disease, asthma, depression, and rheumatoid arthritis.^9^

There is minimal guidance on how Health Canada determines whether the drug targets a disease or condition for which no drug is presently marketed or demonstrates a significant increase in the benefit–risk profile over existing therapies. Eligible drugs must have demonstrated promising clinical effectiveness in clinical trials, possess an acceptable safety profile based on a benefit/risk assessment, and be of high quality. The potential of the drug can be construed from trials with surrogate markers that require validation, Phase II trials that would require confirmation with Phase III trials, or, small to moderately sized Phase III trials requiring confirmation. The improvement in the benefit/risk profile can be satisfied by:

- improving a serious outcome of the condition;
- a favorable effect on a serious symptom or manifestation of the condition for which there is no existing therapy;
- a benefit for individuals unable to tolerate, or are unresponsive to, existing therapies;
- demonstration of effectiveness in combination with other therapies where no information is available or where combined use with existing therapies is not feasible;
- demonstration that the new agent provides benefits that are similar to existing therapies while avoiding serious toxicity present in current therapies and/or avoiding less serious toxicity which results in the discontinuation of treatment of a serious disease; or,
- the ability to provide similar benefit to existing therapies while demonstrating improvement in some factor shown to lead to improved effects on serious outcomes.^10^

NOC/c authorizations can be granted in two circumstances. Either the manufacturer submits a drug evaluation for review, after which Health Canada deems an NOC/c is more appropriate than an NOC, or, the manufacturer can request Advance Consideration under the NOC/c policy prior to filing the submission for review. After deciding to grant an NOC/c, Health Canada will issue an NOC/c-Qualifying Notice (QN), outlining: the additional clinical evidence to be provided in confirmatory studies; post-market surveillance responsibilities; and requirements related to advertising, labelling, or distribution. The sponsor has 30 calendar days to submit responses to the QN, including a draft Letter of Undertaking (LoU). The draft LoU must include: an outline of the confirmatory trials intended to verify the drug’s clinical benefit as well as the proposed timeframe; a provision to submit the required post-market surveillance commitments; a paragraph outlining agreed-upon advertising, labelling, or distribution requirements; a provision to comply with the Notification and Reporting on Specific Issues of Concern requirement; a complete list of ongoing additional clinical trials related to the product; and copies of any market authorizations from any other drug regulatory authority. If the LoU is satisfactory, Health Canada will proceed with issuing an NOC.^11^ Recall that the Food and Drugs Act does not specifically provide for an NOC/c to be issued; instead, under the NOC/c policy, Health Canada issues an NOC/c, but from a legal perspective, it is indistinguishable from a standard NOC.

Drugs under the NOC/c policy are subject to additional post-market surveillance activities, including Adverse Reaction Reporting, Post-Market Surveillance Reporting, Active Surveillance, and Notification and Reporting on Specific Issues of Concern. All serious adverse reactions (ARs) that occur in Canada and all serious unexpected ARs that occurred outside of Canada must be reported to the Marketed Health Products Directorate within 15 days.^12^ For Post-Market Surveillance Reporting, sponsors must, at a minimum, undertake in the LoU to inform the relevant Directorate in writing about the conclusions from the analysis of their annual summary report. This report should indicate whether there has been a significant change in the risk–benefit profile for safe use of the drug. Such changes include ‘change in frequency and or severity of a known risk or the identification of an unknown risk’, with data sources not restricted to the authorized indication. Additional safety information may be required periodically on a case-by-case basis.^13^

Typically, post-market surveillance documents are submitted on an annual basis, although the manufacturer must notify the responsible directorate of a change in the risk/benefit profile without delay. Directorates may also request interim reports, case reports, or issue-related summary reports.^14^ Sponsors may also be required to undertake active surveillance to monitor safety, determined on a case-by-case basis. Finally, sponsors must notify Health Canada, within 15 days, when an expert panel or advisory committee has been struck in a foreign jurisdiction to address an issue related to the product, or when there has been a significant regulatory action in another jurisdiction related to the product. Sponsors must prepare a report on the issue that prompted the action in the foreign jurisdiction and submit it to Health Canada within 30 days of the notification.^15^

Drugs authorized under the NOC/c policy are also subject to enhanced advertising, labelling, and educational material requirements. All advertising material for products authorized under the NOC/c policy must contain boxed text with prominent disclosure of the nature of the market authorization and must be consistent with the specific restrictions or conditions specified in the product monograph (the product label). Sponsors are also requested to receive pre-clearance for all promotional material by the Pharmaceutical Advertising Advisory Board.^16^ NOC/c products are also subject to enhanced labelling requirements, including the requirement to supply the Consumer Information Section/Patient Medication Section and Product Monograph with each NOC/c product. As well, individual labelling restrictions may be required on a case-by-case basis at Health Canada’s discretion. Lastly, all products issued an NOC/c require a Notice of Market Authorization with Conditions, which highlights the nature of the authorization and is communicated through Health Canada’s Health Product InfoWatch.^17^

A sponsor may transfer an NOC/c to an NOC (ie remove the conditions) by submitting the results from one or more confirmatory trials to Health Canada; each set of results may be submitted individually. Health Canada notifies sponsors of the outcome of each submission, but conditions remain in place until Health Canada deems all components of the LoU satisfied. Once the sponsor meets all the conditions of an NOC/c and submits all the documentation, Health Canada removes the designation. If all undertakings are not satisfied or the sponsor foresees an inability to adhere to the agreed upon timelines and/or conditions, the sponsor may submit a new LoU, accompanied by a letter that requests a change in the agreed upon trials and/or extension of the timelines, along with a rationale.^18^

Health Canada has several enforcement powers if a product fails to comply with the LoU in three different circumstances: failure to submit evidence, failure of confirmatory trials to demonstrate clinical benefit, and failure to comply with post-market labelling. First, if a drug manufacturer has failed to submit evidence on or before a day specified by the Minister or if the evidence is not sufficient according to the LoU, the manufacturer cannot continue to market the drug until sufficient evidence is submitted. Second, Health Canada may suspend the NOC/c if the confirmatory trials fail to demonstrate clinical benefit and/or if the confirmatory trials raise safety concerns. Lastly, sponsors may be subject to compliance measure for failure to comply with post-market labelling requirements, including a stop-sale, where required. Health Canada also has the discretion to restrict the patient population or distribution for which the drug was authorized, disseminate further educational material for informed use, or enhance post-market surveillance analysis, on a case-by-case basis.^19^ Additionally, sponsors may voluntarily withdraw authorized indications for any reason, including failure of the confirmatory trial.

Though the name of the NOC/c policy implies that the pathway offers conditional regulatory authorization, arguably, the practical administration of the policy negates its conditional intent. For example, unlike Europe’s conditional regulatory pathway, which automatically expires unless positive action is taken by the manufacturer to renew the authorization, the NOC/c policy requires Health Canada to take action to revoke the authorization. In addition, drugs authorized under the NOC/c policy are granted an NOC, like drugs authorized under the traditional pathway. US research suggests that sponsors view authorization, even with conditions attached, as a ‘vested right’, and therefore not truly conditional on post-market requirements.^20^ Finally, as discussed further below, drugs authorized under the NOC/c policy are only rarely withdrawn for failing to confirm clinical benefit or to comply with post-market requirements. Taken together, these observations suggest that the NOC/c policy is more accurately described as a facilitated regulatory pathway for drugs that addresses unmet medical needs and expedites patient access to new drugs.^21^ Nevertheless, the NOC/c is named to reflect conditionality as the intended purpose, and unlike the FDA (see discussion below), Health Canada has not publicly negated the conditional nature of the NOC/c policy.

In addition to the NOC/c policy, drug manufacturers may apply for Priority Review of drug submissions. To be eligible for Priority Review, the submission must be for a drug that targets a ‘serious, life-threatening or severely debilitating disease or condition for which there is substantial evidence of clinical effectiveness.’^22^ The evidence must demonstrate that the drug treats a disease or condition for which no drug is presently marketed in Canada or provides an improvement in the benefit/risk profile over existing therapies.^23^ If Priority Review status is granted, the submission will be eligible for a shortened review target of 180 calendar days rather than the standard 300 days for non-priority review.^24^ Submissions that request Advance Consideration under the NOC/c policy exclude eligibility for Priority Review status.^25^

Health Canada is currently consulting about reforms to its regulatory approach, including placing terms and conditions on any drug or device authorization.^26^ In 2018, amendments to the Food and Drugs Act made it an offence to fail to comply with terms and conditions placed on therapeutic product authorizations, including drugs, devices, and clinical trial authorizations.^27^ Once the associated regulations are finalized, published, and implemented, these amendments will permit terms and conditions to be added to any drug or device authorized by Health Canada. The amendments were tested during the COVID-19 pandemic via an Interim Order (IO), valid for a one-year term from September 16, 2020, which enabled Health Canada to place terms and conditions on COVID-19 vaccines and testing devices.^28^ Though there is no direct language stipulating that failure to comply with the terms and conditions can lead to withdrawal or amendment of the authorization, it follows that information gathered via the terms and conditions may alter the risk–benefit profile, which could lead to regulatory action. Health Canada amended the Food and Drug Regulations to transition drugs and vaccines authorized under the IO to permanent authorization under the Food and Drug Regulations.^29^ The proposed regulations and accompanying guidance documents indicate that the use of terms and conditions will replace the NOC/c pathway, although the transition pathway for drugs currently authorized under the NOC/c policy is unclear.^30^ The move of the NOC/c policy to regulation should enable Health Canada to take stronger enforcement action in the event of non-compliance.

Analyses of the NOC/c policy to date have generally been limited to small sample sizes or focus on discrete issues. For example, Lexchin has examined 22 NOC/c authorizations between 1998 and 2006, the publication of confirmatory studies required by Health Canada for 31 drugs authorized under the NOC/c policy^31^, the characteristics of pivotal trials for 19 NOC/c authorizations^32^, the relationship between the use of the Priority Review pathway and NOC/c policy and therapeutic gain offered by new products^33^, and post-market safety warnings for 27 NOC/c authorizations.^34^ Law updated Lexchin’s review of the first 22 NOC/c authorizations, including 70 authorizations.^35^ More recently, Andersen reviewed 17 oncology drugs authorized under the NOC/c policy for 22 indications between 2010 and March 2017.^36^

## III. JURISDICTIONAL COMPARISON

Other jurisdictions have introduced life-cycle regulatory mechanisms. In this section, we describe life-cycle regulatory mechanisms in the United States and the European Union, highlighting differences between the jurisdictions and potential lessons from international experience.

The United States Food and Drug Administration (US FDA) introduced the Accelerated Approval (AA) pathway to facilitate the approval of drugs intended to treat serious or life-threatening illnesses with an unmet medical need, for which approval is based on a surrogate endpoint that is reasonably likely to predict clinical benefit or on an intermediate clinical endpoint. For the purposes of AA, a surrogate endpoint is a marker that is thought to predict clinical benefit, but is not itself a measure of clinical benefit, and an intermediate clinical endpoint is a measure of a therapeutic effect that is reasonably likely to predict the clinical benefit of a drug.^37^ The AA pathway is typically used in settings in which the disease course is long and an extended period of time is required to measure the intended clinical benefit of the drug, such as for oncology drugs. To be eligible for AA, the drug must target a serious condition, provide a meaningful therapeutic benefit over any existing treatments, and demonstrate an effect on an endpoint that is reasonably likely to predict clinical benefit.^38^ The same statutory standards for safety and effectiveness as drugs granted traditional approval must be met; for effectiveness, substantial evidence based on adequate and well-controlled clinical investigations is required, and for safety, sufficient information to determine whether the drug is safe for use under conditions prescribed, recommended, or suggested in the proposed labelling is necessary.^39^ Once approved, drug companies are required to conduct studies to verify the surrogate endpoint or intermediate clinical endpoint’s effect on irreversible morbidity or mortality or other clinical benefit.^40^ These trials are required to be completed with due diligence, interpreted to mean they must be conducted promptly to facilitate determination of whether clinical benefit has been verified.^41^ Approval under AA may be withdrawn if a trial fails to verify the predicted clinical benefit, other evidence demonstrates that the product is not shown to be safe or effective, the applicant fails to conduct any required post-approval trial of the drug, or the applicant disseminates false or misleading promotional materials relating to the product. If the required post-market confirmatory trials fail to confirm clinical benefit and FDA determines that a drug should be withdrawn, the drug manufacturer can agree to withdraw it or request a public hearing.^42^ Like the NOC/c policy, the AA pathway falls short of being a conditional regulatory pathway. Indeed, FDA officials have distinguished AA from other international examples of conditional authorization, including that of the European Union.

Several analyses have critiqued the AA pathway.^43^ The AA pathway has been criticized^44^ for its reliance on surrogate endpoints, strength of evidence developed in post-market trials, delays in post-market trials, difficulty enforcing post-market studies due to lack of legislative authority, lack of political will, and pricing challenges that result from approving drugs with significant clinical uncertainties.^45^ While not immune from challenges, the AA pathway is enshrined in the Code of Federal Regulations, providing US FDA with clear scope, eligibility criteria, and withdrawal procedures.^46^ Additionally, greater transparency exists regarding drugs authorized under the AA pathway compared to drugs authorized under the NOC/c policy, including timely and accessible publication of authorizations and the conditions of authorization. For example, meetings of the Oncologic Drugs Advisory Committee are publicly accessible. In April 2021, meetings were held over the course of three days to discuss several drugs authorized under the AA pathway that subsequently failed their confirmatory trials. Anyone could attend the meeting to hear the discussions and decisions, and members of the public could submit questions or comments.^47^

Like the NOC/c policy in Canada, the AA pathway has recently been reformed, on December 29, 2022, by the Consolidated Appropriations Act for 2023, containing the Food and Drug Omnibus Reform Act (FDORA). In addition to providing funding to FDA and reauthorizing the user fee program, FDORA introduced changes to the AA pathways, including: authorizing FDA to require a post-approval study or require studies to be underway prior to or within a specific timeframe of receiving AA; authorizing FDA to require status reports every six months on the progress of confirmatory trials; streamlining the withdrawal process for a drug that fails or does not make progress on its confirmatory trial; requiring FDA to develop guidance on using novel endpoints and study designs in confirmatory trials; and requiring FDA to establish an internal coordinating council to advise on the AA program.^48^

The Fast Track designation, Breakthrough Therapy designation, and Priority Review designation are also available to expedite the authorization of certain drugs for serious conditions. The Fast Track designation is intended for drugs for the treatment of a serious or life-threatening disease or condition that demonstrates the potential to address unmet medical needs. Drugs with Fast Track designation benefit from rolling review and frequent interactions with the review team, including meetings with the Food and Drug Administration. The Breakthrough Therapy designation is for drugs intended to treat a serious or life-threatening disease or condition where there is preliminary clinical evidence indicating that the drug may demonstrate substantial improvement on a clinically significant endpoint over available therapies. If eligible, drugs with Breakthrough Therapy designation benefit from rolling review, intensive guidance on an efficient drug development program, including advice on clinical trial design, organizational commitment to involve senior managers and experienced project management staff for a proactive, collaborative, cross-disciplinary review. Finally, the Priority Review designation is available for drugs for serious or life-threatening conditions that: if approved, would provide a significant improvement in safety or effectiveness, have been designated as a qualified infectious disease product, or any application or supplement for a drug submitted with a priority review voucher. Drugs that qualify for Priority Review benefit from a shorter timeline for the review of the marketing application (6 months compared to 10-month standard review). Drugs may be eligible for more than one of the four above designations/pathways.^49^

In the European Union, the European Medicines Agency supports the development of medicines to address unmet medical needs through conditional marketing authorizations (CMAs). To be eligible for CMA, the Committee for Human Medicinal Products (CHMP) must find that: the benefit of immediate availability outweighs the risk of less comprehensive data; it is likely that the applicant will be able to provide the comprehensive data; unmet medical needs will be fulfilled; and the benefit–risk balance of the product is positive. The CMA Regulation applies to medicinal products, which treat, prevent, or diagnose seriously debilitating diseases or life-threatening disease, medicinal products to be used in emergency situations or in response to public health threats, and medicinal products designated as orphan medicinal products. Holders of a CMA are required to complete ongoing studies, or to conduct new studies to confirm that the risk–benefit balance is positive. CMA products are also subject to additional labelling and advertising requirements, as well as submitting periodic safety update reports every six months. Unlike in Canada, the timelines for studies must be clearly specified, and the obligations and timelines must be made publicly available. Each CMA is valid for one year and must be renewed annually if the obligations are not satisfied. Once the obligations have been fulfilled, the CHMP may grant a full marketing authorization.^50^

The CMA scheme is distinct from marketing authorizations granted in exceptional circumstances (EC). CMAs are granted where it is expected that the data lacking from the initial submission will eventually be provided, whereas EC authorizations are granted where it will never be possible to assemble comprehensive data on the efficacy and safety under normal conditions of use, either because the condition to be treated is rare or because collection of full information is not possible or is unethical. EC authorizations are reviewed annually to reassess the risk–benefit analysis, but the authorization is valid for five years, after which time a renewal must be granted which will be for an unlimited time. Like CMAs, specific procedures or obligations may be imposed as part of the marketing authorization, but the fulfillment of such obligations may not ever result in a normal marketing authorization. Applicants are required to submit a statement indicating that the applicant is unable to provide comprehensive non-clinical or clinical data under normal conditions of use, a list of all the data that cannot be provided, justifications on the grounds for approval under EC, and proposals for detailed information on the specific procedures or obligations to be conducted. Even if the applicant does not provide this information, the CHMP may propose approval under EC.^51^

Like AA in the United States, CMA has been comprehensively analyzed.^52^ For example, the operation of the CMA Regulation has been criticized for authorizing drugs with lower evidence rather than enabling the marketing of promising new medications and serving as a de factor regulatory backstop for drug submissions that do not reach the standard regulatory evidence threshold.^53^

## IV. ANALYTICAL APPROACH TO CANADA’S NOC/C PATHWAY

We collected and analyzed data on all drugs authorized under the NOC/c policy in Canada from 1998 to the end of 2021. We included all NOC/c authorizations, including authorizations for new active substances, new indications for already authorized substances, and generics. We first compiled a comprehensive list of all drugs authorized under the NOC/c policy by searching multiple databases, because Health Canada does not maintain a single, up-to-date list of all drugs authorized under the NOC/c policy. We searched the publicly available list of drugs on Health Canada’s NOC/c webpage^54^, the Notice of Compliance database^55^, the Drug Product Database^56^, Health Canada’s Drug and Health Product Submissions Under Review Database^57^, and archived versions of Health Canada’s NOC/c webpage from the Wayback Machine, a digital archive of internet webpages.^58^ The Wayback Machine permitted us to include NOC/c information that was previously posted on Health Canada’s websites but has since been deleted or removed. From these sources, we identified the name of the authorized drug, the date of authorization, the date of removal of conditions (if applicable), and the authorized indication.

We also collected QNs, where available, from the NOC/c webpage or from Google searches, and Regulatory Decision Summaries (RDS) and Summary Basis of Decisions (SBD) Health Canada’s Drug and Health Product Register^59^ to identify information about the regulatory timeline, evidence submitted to support authorization, reasons for granting the NOC/c, and post-market confirmatory trials. We also reviewed SBDs and RDSs to determine the justification for authorization under the NOC/c policy. Reasons for authorization under the NOC/c policy were not reported in a standardized fashion. Instead, we reviewed the entirety of the document and identified where a deficiency in the pre-market evidence was described, either specifically in relation to authorization under the NOC/c policy or more generally. We did not assess the characteristics of pivotal trials and make judgments about whether uncertainties were present or not.

We searched clinicaltrial.gov to identify clinical trials and confirm the status of confirmatory trials for indications with active conditions, because most clinical trials for Canadian regulatory review are registered on this site to meet Health Canada registration requirements. Published literature linked to clinicaltrial.gov identifiers was also reviewed for additional information about the pre- and post-market clinical trials. We also reviewed product monographs from the Drug Product Database online query to confirm identification of pivotal and post-market trials. The enrollment, study design, and endpoints of pre- and post-market clinical trials were identified first through SBDs or RDSs. If those were not available, information was gathered from clinicaltrials.gov.

## V. WHAT ANALYSIS OF CANADA’S NOC/C PATHWAY REVEALED

### V.A. Characteristics of NOC/c Authorizations

We identified 148 drug-indication authorized between 1998 and 2021 (See Appendix 1). As of June 2022, 76 (51%) have successfully had the conditions removed and the authorization transferred to a standard NOC. 50 (34%) had active conditions still in place. We categorized the remaining 22 (15%) as other, which included: post-market cancellations (1), suspensions (3), indication or product withdrawals (5), discontinuations (6), and a class of memantine products for which the conditions were removed for ethical reasons (7). 55/148 authorizations were not new substances. That is, the drug had previously been authorized for another indication, either as an NOC/c or an NOC. For the remaining 93, the NOC/c was the first authorized indication for the drug.

A large majority of NOC/c authorizations are indicated for cancer. About, 72% (107/148) of all NOC/c authorizations included in our sample are classified as antineoplastic and immunomodulating agents according to Anatomical Therapeutic Chemical (ATC) Classification, first level.^60^ The remaining 28% are a mix of various other ATC groups, including anti-infectives, nervous system, alimentary tract, blood and blood forming agents, musculo-skeletal, cardiovascular, sensory organs, antiviral, various, and unassigned (See Table 1).

**Table 1:** ATC Classification of NOC/c Authorizations, 1998–2021

ATC classification, first level	Conditions removed	Active	Other	Total
Antineoplastic & immunomodulating agents	56	42	9	**107**
Anti-infectives for systemic use	12		1	**13**
Nervous system	3	9		**12**
Alimentary tract and metabolism		1	4	**5**
Various	3			**3**
Blood & blood forming organs	2			**2**
Musculo-skeletal system		2		**2**
Cardiovascular system		1		**1**
Antiviral			1	**1**
Sensory organs			1	**1**
Unassigned			1	**1**

Of the 148 authorizations, 27 were identified as being authorized after receiving Advance Consideration. Five were identified as eligible for expedited review under Priority Review. Upon a strict reading of the NOC/c Guidance Document and the Priority Review Guidance Document, it should not be possible for a drug to be authorized under both pathways; the Priority Review pathway requires substantial evidence of clinical effectiveness, while the primary feature of the NOC/c pathway is that it permits discretion to authorize drugs based on ‘promising evidence’ of clinical effectiveness.^61^ Therefore, if a drug meets the eligibility criteria for Priority Review status, it should be reviewed under the standard regulatory pathway. Whether or not a drug received Advance Consideration or Priority Review status was inconsistently reported, however, so these findings are not necessarily indicative that the remaining authorizations in the sample did not receive Advance Consideration or Priority Review status.

For authorizations that had fulfilled the conditions and been transferred to an NOC (*n* = 76), the average length of time that conditions were in place was 1250 days, or approximately three and a half years, with a range from 63 to 4505 days. The median length of time was 1023 days, or a little under three years. This is shorter than analyses from the EU, which found a median time of five years to switch to regular marketing authorization.^62^

Even though analysis of authorizations with conditions still active is skewed by recent authorizations, as of June 15, 2022, the average length of time on market for authorizations with active conditions is 1265 days, or approximately three and a half years, with a maximum length of 5606 days, or 15 years.^63^ Of the 50 authorizations with active conditions, exactly half (25/50) have been on market for at least three years without fulfilling the post-market conditions.

### V.B. Pre-market Evidence

Of the 148 authorizations, we were able to identify the characteristics of the pivotal trials for 99 authorizations. Most (73.7%, 73/99) of the authorizations were based off only one pivotal trial. The average enrolment in pivotal trials was 655; however, the median enrollment was 322. 46 (46.5%) were single-arm trials, while 38 (38.4%) were randomized design, either parallel, crossover, or sequential assignment. Most were either Phase II (42/99, 42.4%), Phase III (27/99, 27.3%), or Phase II/III (3/99, 3.0%) trials; however, a not insignificant amount were Phase I, I/II, or I/III (22/99, 22.3%). Response rate (objective or other) was the most commonly reported endpoint for pivotal trials (79/99, 78.8%). Overall survival (OS), the gold standard for demonstrating clinical benefit^64^, was reported in only one pivotal trial. Almost three-quarters (71/99, 71.7%) of the pivotal trials were open label. Finally, approximately half (53/99, 53.5%) of the trials included no comparator; of those that did have a comparator, half used a placebo and half used other drugs (Table 2).

**Table 2:** Pre-Market Pivotal Trial Characteristics, 1998–2021 (*n* = 99)

Number of pivotal trials	1 2 3+	73 19 7	73.7% 19.2% 7.1%
Enrollment Mean: 654.9 Median: 322 Range: 28–9366	< 250 250–500 > 500	40 27 30	40.4% 27.3% 30.3%
Study design	Single arm Randomized Parallel assignment Crossover assignment Sequential assignment Multiple Non-randomized, parallel assignment	46 38 33 4 1 10 3	46.5% 38.4% 33.5% 4.0% 1.0% 10.1% 3.0%
Phase	Phase I Phase I/II Phase I/III Phase II Phase II/III Phase III	5 16 1 42 3 27	5.1% 16.2% 1.0% 42.4% 3.0% 27.3%
Primary endpoint	Objective response rate Other response rate Disease-/progression-free survival Time to endpoint OS Other	43 35 14 3 1 3	43.4% 35.4% 14.1% 3.0% 1.0% 3.0%
Masking	Open-label Not open-label Double Triple Quadruple Multiple	71 25 8 4 8 5	71.7% 25.3% 8.1% 4.0% 8.1% 5.1%
Comparator	None Placebo Other drug(s) Other drug(s) and placebo	53 21 21 2	53.5% 21.2% 21.2% 2.0%

For pre-market uncertainties, we categorized uncertainties into seven categories: study design, sample size, population characteristics, short-term data, dosing, OS, and safety uncertainties. Study design uncertainty refers to pivotal trials that were single arm or lacked a comparator, used a surrogate endpoint, or if the pivotal trial(s) were Phase I or II. Sample size uncertainties were present where the evaluable data from the pivotal trial(s) had small patient populations. Population characteristic uncertainties refer to circumstances where specific patient characteristics, such as age, gender, ethnicity, biomarker status, or comorbidity, were under- or unrepresented in the pivotal trial(s). Short-term data uncertainties are where either the duration of the pivotal trial was short, or the authorization was based on interim results from an ongoing trial. Dosing uncertainties exist where the pivotal trial(s) did not establish the appropriate dose. OS uncertainty exists if OS was not an endpoint in the pivotal trial(s), or the trial was still ongoing, and sufficient data had not yet been collected to determine OS. Lastly, safety profile uncertainty indicates that there is outstanding uncertainty about the severity and incidence of adverse events.

The average authorization mentioned 2.64 uncertainties. The most commonly mentioned uncertainties at the time of authorization were study design (57/99, 58%) and lack of confirmed OS benefit (55/99, 56%). Short-term data and population characteristic were the next most frequently mentioned uncertainties, identified in 40 (40%) and 41 (41%) authorizations, respectively. Safety profile uncertainties were present in 31 (31%) authorizations, and sample size uncertainties were present in 26 (26%). The least common uncertainty was dosing, which was present in 11 (11%) authorizations.

### V.C. Post-market Confirmatory Trials

Of the 148 authorizations, there was information about the post-market trials for 107 authorizations. A total of 227 trials were reviewed. Almost half (49/107, 45.8%) of the authorizations had only one identified confirmatory clinical trial. 25 (23.4%) had 2 identified confirmatory clinical trials, and 33 (30.8%) had 3 or more identified confirmatory clinical trials. Enrolment across all trials per authorization varied significantly. The average enrolment per authorization was 1302.1, and the median was 743. Total enrolment ranged from 30 to 9366 (See Table 3).

**Table 3:** Post-Market Trials (Pooled) by Authorization (*n* = 107)

Number of trials	1 2 3+	49 25 33	45.8% 23.4% 30.8%
Enrollment Mean: 1302.1 Median: 743 Range: 8–9366	< 250 250–499 500–1000 1000+	19 11 34 43	17.8% 10.3% 31.8% 40.2%

Looking at the trials individually provides additional insight on the characteristics of post-market confirmatory trials. Of the 227 identified post-market trials, average enrolment was 625.6, with a median of 327. Almost half (109/227, 48%) were Phase III trials. 45 (19.8%) were Phase I or I/II, 64 (28.2%) were Phase II or Phase II/III, and 4 (1.8%) were Phase IV. More than half (128/227, 56.4%) were randomized. 60 (26.4%) were single arm, 23 (10.1%) were non-randomized, multiple-arm trial designs, and 2 (0.9%) were observational. More than three-quarters (166/227, 78.3%) of the trials were open-label. Of the 54 (23.8%) that were masked, most were either double (23/54, 42.5%) or quadruple (28/54, 51.9%) masked.

A wide range of endpoints were reported as the primary endpoint in the post-market trials. Response rate (objective or other) was the primary endpoint in 77 (33.9%) post-market trials. Progression-free survival was the next most common endpoint, reported in 47 (20.7%) post-market trials. OS was reported as the primary endpoint in only 17 (7.5%) post-market trials. However, when we included secondary endpoints, 113 (49.8%) reported OS as a primary or secondary endpoint (See Table 4).

**Table 4:** Post-Market Trial Characteristics by Trial (*n* = 227)

Enrollment Mean: 625.6 Median: 327	< 250 250–499 500–1000 1000+	89 52 47 35	6.8% 13.1% 22.9% 57.2%
Study design	Single arm Randomized Parallel assignment Crossover assignment Sequential assignment Non-randomized, multiple arms Observational	60 128 119 7 2 23 2	26.4% 56.4% 52.4% 3.1% 0.9% 10.1% 0.9%
Phase	Phase I Phase I/II Phase II Phase II/III Phase III Phase IV Other	30 15 60 4 109 4 6	13.2% 6.6% 26.4% 1.8% 48.0% 1.8% 2.6%
Primary endpoint	Other response rate Progression-free survival Objective response rate Pharmacokinetics OS Scale, score, other value Safety Dosage Disease-, recurrence-, event-free survival Time to endpoint Duration of response	50 47 27 18 17 17 16 15 12 5 1	22.0% 20.7% 11.9% 7.9% 7.5% 7.5% 7.0% 6.6% 5.3% 2.2% 0.4%
Masking	Open-label Masking Single Double Triple Quadruple	166 54 1 23 2 28	78.3% 23.8% 0.4% 10.1% 0.9% 12.3%
Comparator	None Placebo Other drug(s) Other drug(s) plus placebo	108 31 71 12	47.6% 13.7% 31.3% 5.3%

We also looked at the start date of post-market trials. For 174 post-market trials, there was an identifiable study start date. Of those, 159 started prior to the NOC/c authorization date.

## VI. LESSONS FROM EXPERIENCE TO DATE WITH THE NOC/C PATHWAY

The NOC/c policy has provided over 20 years of experience with a life-cycle regulatory authorization pathway. To our knowledge, our analysis is the most comprehensive review of NOC/c authorizations to date. Lessons derived from our analysis are useful for informing future regulatory reforms currently underway in Canada and internationally, including the use of terms and conditions and other proposed reforms. Based on our review of experience to date with the NOC/c policy, as well as other reported analyses of life-cycle regulatory pathways, we make the following recommendations for consideration as regulators move toward a life-cycle approach to drug and device regulation: increasing decision-making transparency, selecting appropriate health technologies, setting clear and comprehensive pre-market evidence requirements, designing appropriate post-market confirmatory trials, and enhancing enforcement and compliance. We discuss each recommendation in turn.

### VI.A. Increasing Decision-Making Transparency

Generally, drugs authorized under the NOC/c policy are based on less evidence of safety and clinical efficacy than regular drug authorizations, and therefore drugs authorized under the NOC/c policy may pose greater risks to patients. This is supported by our findings that most NOC/c authorizations are based on one single-arm trial rather than the standard regulatory threshold of ‘substantial evidence’, which has been interpreted as generally requiring two randomized controlled trials (RCTs).^65^ Additionally, while the NOC/c policy was initially introduced to permit authorization of drugs addressing unmet medical needs where clinical benefit still needs to be confirmed, our textual analysis of RDSs and SBDs demonstrates that there are several different types of evidentiary uncertainties identified at the time of authorization, which may influence patient and physician decision-making in different ways than needing to confirm clinical benefit in a larger, randomized trial. For this reason, transparency in decision-making is crucial to ensure stakeholders are appropriately informed of the evidentiary uncertainties that were identified at the time of authorization. Transparency also promotes regulatory accountability.

Lack of transparency has been widely cited as a significant barrier to meaningful analysis of drug authorizations in Canada and internationally.^66^ Though transparency is important and encouraged for all drug authorizations, the heightened risks associated with drugs authorized under life-cycle regulatory pathways warrant proactive transparency about the nature of uncertainties at the time of initial authorization, the nature and status of post-market requirements, and decision-making and analytic frameworks employed in on-market assessments. Currently, information about drugs authorized under the NOC/c policy in Canada is published across different databases and is often outdated or inconsistent. There is little information made public about the process of regulatory decision-making, which makes it difficult to assess the reasoning behind decisions. Specifically, pre-market uncertainties are reported inconsistently, which makes it difficult to ascertain why a drug was authorized under the NOC/c policy rather than granted a standard NOC and how those uncertainties will be resolved in the post-market setting. Health Canada should therefore make information about new drug authorizations transparent at the time of the authorization, as well as ongoing transparency about the status of post-market commitments, and any subsequent decisions. Increased transparency of decisions, and the rationales behind decisions, will contribute to increased accountability and consistency in decision-making.

Several recent initiatives by Health Canada have increased transparency in decision-making for authorized drugs, including the public release of clinical information (PRCI) portal and publication of regulatory documents, including SBDs and RDSs. Health Canada uses the PRCI portal to proactively release all clinical information once a final regulatory decision is made.^67^ In addition, Health Canada publishes SBDs and RDSs on its website for eligible products. SBDs are published following the initial regulatory authorization of a drug, and RDSs are published following subsequent regulatory decisions. Both documents typically include some information about the regulatory decision, the scientific rationale for the decision, and post-authorization activity.

Despite the above initiatives, transparency in decision-making processes continues to be opaque, including how drugs are deemed eligible for authorization under the NOC/policy, and how decisions are made to remove conditions and transfer the authorization to an NOC approval. For example, not all QNs include an explicit statement on the potential for withdrawal of the authorization based on confirmatory trials. Even where language is included, different thresholds for withdrawing conditions are implied by the inconsistent use of language, creating confusion about the requirements for removing conditions (See Table 5). Second, available documents and information are inconsistently available. For example, post-authorization activity tables (PAAT), a section available on SBDs intended to provide up-to-date information on regulation activity, are not frequently updated. Many remain two years or more out of date, limiting their utility in understanding the authorization process and informing clinical decision-making. Even where these documents are available, Health Canada inconsistently reports relevant information needed to enable stakeholders to make informed decisions and promote accountability for decision-making. While Health Canada has participated in international initiatives to standardize benefit–risk assessment processes to increase transparency, subsequent reviews have demonstrated that Health Canada does not publish many of the identified components, including outstanding issues, regulatory history, summary of risks, weighting and valuing benefits and risks, and others.^68^ Proactive disclosure of this information supports accountability under life-cycle regulatory pathways.

**Table 5:** Examples of Withdrawal Language for NOC/c Authorizations

‘Seattle Genetics, Inc. is aware that the indications for Adcetris ([Hodgkin’s lymphoma] and/or [systemic anaplastic large cell lymphoma]) can be withdrawn if either or both studies are unsuccessful.’ (Brentuximab vedotin, 2/1/2013) ‘If the proposed confirmatory studies fail to demonstrate OS benefit the glioblastoma indication should be voluntarily withdrawn.’ (bevacizumab, 3/24/2010) ‘Janssen Inc. should acknowledge that the indication authorized under the NOC/c pathway for BALVERSA under control #224529 can be withdrawn or revised if Study [trial identifier removed] does not demonstrate an improvement in efficacy, compared with Vinflunine or Docetaxel or Pembrolizumab that is both statistically and clinically significant.’ (erdafitinib, 10/25/2019) ‘Pfizer has indicated that it will withdraw the indication should the primary endpoint of the Phase 3 trial not reach statistical significance.’ (Palbociclib, 3/16/2016) ‘Acknowledge that the indication for IMFINZI may be withdrawn if study D419BC00001 does not demonstrate that IMFINZI monotherapy is associated with an overall survival (OS) benefit that is both statistically and clinically significant (compared to standard of care).’ (durvalumab, 11/3/2017) ‘Acknowledge that the indication authorized under the NOC/c pathway for Lartruvo under control number 203478 can be withdrawn if the results from Study JGDJ do not demonstrate a positive benefit/risk profile as determined by BGTD.’ (olaratumab, 11/23/2017) ‘Acknowledge that the indication for [primary progressive multiple sclerosis] PPMS may be withdrawn in the event of lack of clinical benefit of OCREVUS in patients with PPMS in study WA40404.” (ocrelizumab, 2/14/2018)

As discussed above, life-cycle regulatory authorization pathways have the potential to increase risks to patients by authorizing drugs that have not yet met the standard regulatory threshold of substantial evidence of clinical effectiveness as demonstrated by two well-controlled trials. As such, eligibility for authorization under life-cycle pathways should be limited to circumstances where the benefits of early access outweigh the risks of clinical effectiveness and safety uncertainties. This may require reworking the definition of ‘substantial evidence’ and moving away from the current separation of ‘substantial’ and ‘promising’ evidence based on the number and type of clinical trial alone. Adopting a more flexible definition of substantial evidence requires transparency about pre-market evidence uncertainties and the capacity for additional research to address those uncertainties.

In sum, as health regulators move toward life-cycle regulatory approaches, which require contextual pre-market evidence assessments, greater transparency is required. Specifically, transparency about what evidence is submitted to support regulatory authorization, about the outstanding uncertainties identified at the time of authorization, the terms of the authorization, eligibility determinations, status of post-market conditions, and determinations to remove conditions. Such transparency is necessary to safeguard regulatory accountability and enable stakeholders, including physicians, patients, and reimbursement decision-makers to make informed decisions. While there are existing tools to encourage standardized pre-market assessments, they currently are not being used by Health Canada. Measures should be adopted to encourage and ensure high levels of transparency across the drug life cycle.

### VI.B. Increased Guidance to Select Appropriate Health Technologies

The NOC/c policy was developed in 1998, after a similar pathway was adopted in the United States following pressure from HIV/AIDS patient advocates to access experimental drugs for this serious, life-threatening disease. Today, the policy is used almost exclusively for oncology drugs, but its eligibility criteria are broad. Cancer, like HIV/AIDS, is explicitly listed as an eligible disease, but the breadth in application of the eligibility criteria has raised concerns that the NOC/c policy is being overutilized and misused. The stated purpose of the NOC/c policy is to facilitate earlier access to promising new drugs. However, previous research has demonstrated that Health Canada is not able to reliably predict which drugs will offer major therapeutic gain.^69^

To justify the heightened risk associated with life-cycle authorizations, eligibility should be limited to circumstances in which the benefits of earlier access outweigh the risks. Most jurisdictions limit life-cycle authorizations to drugs that address an unmet medical need, typically defined as a condition for which no treatment exists, or where the drug provides a significant improvement in the benefit–risk profile over existing therapies. However, there is no widely accepted definition of unmet medical need.^70^ If unmet medical needs remain an eligibility criterion under current regulatory reform efforts, it requires a clear and comprehensive operational definition.

We previously analyzed a cohort of 33 oncology drugs authorized under the NOC/c policy. Our analysis demonstrated that interpreting and applying the eligibility criteria stated in the NOC/c Guidance Document was inconsistent, potentially resulting in overinclusion of health technologies not intended to be captured by the policy. Specifically, we were unable to identify a clear definition of ‘existing therapies’ or required quantum of improvement over existing therapies required to assess the eligibility criteria.^71^ Many jurisdictions also include drugs that provide an improvement over existing therapies as eligible for authorization under life-cycle regulatory authorization. The interpretation of this requirement has proven challenging. For example, it is unclear whether ‘existing therapies’ include off-label drug use, non-pharmaceutical treatment modalities (eg surgery), or treatments available through compassionate access programs. The threshold for demonstrating improvement should be clearly stated. Often, qualifiers such as ‘significant’ or ‘substantial’ precede the requirement to demonstrate improvement. Without defining significant or substantial, regulators are left with a large amount of discretion to determine eligibility.^72^ Both criteria of unmet medical need and improvement over existing therapies may require contextual assessments to adequately reflect clinical realities of the disease and treatment modalities. To allow flexibility, requirements for demonstrating unmet medical need and improvement over existing therapies could be set by a panel of independent experts prior to regulatory review specific to the context of the authorization.

As Health Canada adopts the use of post-market terms and conditions more broadly for all drugs and devices, experience suggests the adoption of clear eligibility criteria to promote appropriate use of the pathway. Authorizations under the NOC/c policy, or other similar life-cycle regulatory pathways, should be used when potential benefits of early access outweigh the risks, and post-market data collection is likely to address the clinical and/or safety uncertainties in a reasonable time period. Well-defined eligibility criteria will provide certainty to drug developers, protect patients, and maintain trust in drug and device regulation.

### VI.C. Setting Clear and Comprehensive Pre-market Evidence Requirements

Life-cycle regulatory pathways enable the authorization of health technologies earlier in the development cycle. In application, the pathways lower the traditional ‘substantial evidence of effectiveness’ standard applied by Health Canada, which generally requires two, well-controlled clinical trials.^73^ The NOC/c policy permits flexibility in deviations from this standard. A drug may be authorized under the NOC/c policy based on clinical trials using surrogate markers that still require validation, Phase II trials, or small Phase III trials. Further, Health Canada has discretion to consider single trials, literature reviews, expert opinions, panels, or pharmacokinetic/pharmacodynamic studies.^74^ Our findings demonstrate that under the NOC/c policy, many drugs have been authorized over the last few decades before meeting the standard regulatory threshold of substantial evidence, requiring two well-controlled trials.

Our findings are consistent with studies from other jurisdictions that have demonstrated an increasing reliance on small, single-arm studies for regulatory authorization in the United States. A 2017 review of 22 drugs authorized under the AA pathway in the United States between 2009 and 2013 found that nonrandomized, noncomparative single-arm studies supported the authorizations of 14 authorizations and 12 authorizations were based on preauthorization studies with fewer than 200 participants.^75^ Similarly, a 2018 review of 64 hematology and oncology drugs and biologics authorized under AA found that 72% were based on single-arm trials, with an overall median efficacy population of 143 patients.^76^ Even outside of alternative regulatory pathways, reliance on single-arm trials has become the norm; 80–85% of oncology drug authorizations are based on a single pivotal trial.^77^ These results are consistent with findings that regulators are increasingly tolerant of uncertainty at the time of authorization.^78^

In addition to the type and size of clinical trials, there is concern about the reliance on surrogate endpoints for drugs authorized under the NOC/c policy and other life-cycle regulatory pathways. Reliance on surrogate markers is often permitted in lieu of validated endpoints, such as OS, to hasten the drug development and regulatory review processes and enable patient access. However, concerns have been raised about overreliance on surrogate endpoints, specifically that they may not accurately predict clinical benefit.^79^ The use of surrogate endpoints has been studied in the United States. A 2009 Government Accountability Office identified 90 drugs authorized based on surrogate endpoints under the AA pathway between 1992 and 2008, as well as 69 drugs authorized under the traditional authorization process based on surrogate endpoints. At the time of the report, FDA did not have specific criteria for deciding when surrogate endpoints would be accepted.^80^ Since then, FDA has published a table of surrogate measures it has used and may accept for regulatory use as required by the 21^st^ Century Cures Act of 2016.^81^ One review of surrogate measures in the table for breast cancer found that none of the accepted surrogates were strongly correlated with OS.^82^

Our study is the first comprehensive assessment that includes identification of surrogate endpoints for drugs authorized under the NOC/c policy, demonstrating that 95% of NOC/c authorizations included in our sample were authorized based on surrogate endpoints. Response rate and disease- or progression-free survival were the most used endpoints. Our findings confirm a previous study reviewing a sample of 22 NOC/c oncology authorizations, finding that all authorizations were based on a single-arm trial or a trial using a surrogate endpoint.^83^ Despite literature demonstrating that surrogate endpoints may not predict clinical benefit^84^, this did not appear to be a consideration in the regulatory review process. Health Canada did not explicitly differentiate between validated or non-validated surrogate endpoints. Health Canada does not publish a list of accepted surrogate endpoints for authorization under the NOC/c policy or under standard authorization.

Lowering the threshold for market entry may erode public trust in regulators and has downstream implications for other decision makers in the health system, including payors, healthcare providers, and patients. Additionally, it may result in the authorization of some drugs that prove to be only modestly effective or not effective at all. To mitigate this, increased transparency about pre-market evidence assessments, including which surrogate endpoints are suitable for regulatory decision-making based on current evidence, would improve consistency and accountability. The relevance of flexible pre-market evidence assessments under regulatory reforms remains to be seen. The Notice of Intent suggests that the new terms and conditions paradigm is not intended to permit submissions not meeting the ‘substantial evidence’ threshold to gain market authorization. Removing the option for drugs to be authorized prior to reaching that threshold may have significant implications for the ability for certain drugs to gain market authorization. Instead, a more flexible, yet structured, approach to determining substantial evidence, as discussed above, should be adopted to promote accountability.

### VI.D. Designing Appropriate Post-Market Clinical Trials

Post-market confirmatory trials are a crucial component of life-cycle authorization mechanisms. However, the design of post-market studies raises concerns, specifically, the use of surrogate endpoints, lack of randomization, and determining a threshold for meaningful change in endpoints, all of which contribute to uncertainty at the time of initial authorization and upon completion of post-market trials.^85^ Confirmatory trials need to be designed to address uncertainties identified at the time of the authorization and to meet specified timelines. However, experience in other jurisdictions suggests that post-market evidence does not always accumulate to inform regulatory status.^86^

The first concern is the failure to replace evidence from surrogate endpoints with evidence from validated endpoints.^87^ One study previously found that for many drugs authorized under AA, the surrogate endpoints used in the confirmatory trials were the same as those used in the pivotal trials.^88^ Our findings confirm previous research demonstrating that most confirmatory trials utilize surrogate endpoints.^89^ Furthermore, 63.6% (63/99) of authorizations used the same surrogate endpoint as the primary endpoint in the confirmatory trial that was used in the pivotal trial(s). Looking at the individual trial level, 33.9% (77/227) of post-market trials used the same primary endpoint at the corresponding pivotal trial. While OS is not always the ideal or appropriate endpoint, surrogate endpoints such as response rate and progression-free survival are more reliable in some indications than others^90^, and it is currently unclear whether Health Canada considers the appropriateness of surrogate endpoints in post-market trials. In fact, currently there are no requirements regarding the design of confirmatory trials, including acceptable endpoints.

Another concern about post-market trials is the lack of randomization. While post-market randomized trials can face practical and ethical challenges, the lack of randomization limits the ability to determine the effectiveness of the drug.^91^ Currently, confirmatory trial design is largely determined by the manufacturer, based on already ongoing clinical trials. Because most post-market trials were ongoing at the time of authorization, there may be little opportunity under current regulatory processes to amend or alter the design of post-market trials. Additionally, 48/99 (48.5%) NOC/c authorizations for which pre- and post-market trial data were available used a post-market trial that was also used to provide pre-market evidence. This similarly represents limited opportunity to ensure that post-market trials are designed to resolve uncertainties. Additionally, most (93%, 211/227) post-market confirmatory trials are industry sponsored, though for some rare diseases, post-market activities include evidence generated from patient registries. Reliance on industry-sponsored clinical trials raises transparency and accountability concerns and demonstrates a lack of systems readiness for the use of real-world data, or data collected routinely from health care.^92^ The use of real-world data generated by pragmatic clinical trials, patient registries, or other mechanisms would require additional administrative capabilities and infrastructure, as well as mechanisms to address the issues that arise from the overlapping jurisdiction of provincial and federal governments. However, lack of transparent mechanisms and processes to ensure that post-market trials are appropriately designed limits the ability of regulators and other stakeholders to assess clinical benefit, including randomization, but also in relation to trial design more generally.

Together, the above raises serious concerns about the current appropriateness of post-market confirmatory trials and whether they can improve the pre-market evidence base. The use of the same surrogate markers as the pivotal trials, lack of randomization, and the inability to ensure that post-market trials address evidentiary uncertainties at the time of authorization undermine the promise of life-cycle regulatory approaches to confirm clinical benefit post-market. Based on our review of confirmatory trials, the post-market trials do not generally improve on pre-market ones. Concerns arise because a drug authorized under the NOC/c policy is indistinguishable from a drug authorized under the standard regulatory pathway. Unless a motivated patient, physician, or pharmacist makes an effort to review the regulatory history of the drug, evidentiary deficiencies remain opaque. After the conditions are removed, any mention of authorization under the NOC/c policy is removed from the product monograph, which remains the primary method of communication about safety and efficacy of authorized drugs.

Positive steps have been made toward increased post-market oversight of drugs that can help to address uncertainties that arise post-market. In 2014, Vanessa’s Law was passed, amending the Food and Drugs Act to enhance transparency and accountability through improvements to the post-market oversight of drugs and medical devices.^93^ Most notably, Vanessa’s Law led to the implementation of the Public Release of Clinical Information database, which publishes clinical information after a final regulatory decision. It further empowers the Minister to order a person to provide information in their control if there is a belief that the product presents a serious risk of injury, or to conduct assessments, tests, studies, or monitoring.^94^ According to the published guidance, these powers are intended to be used as a last resort, only if the parties are unwilling to comply voluntarily.^95^ The impact of these amendments remains to be seen. The amendments and accompanying guidance documents clarify Health Canada’s authority to require the submission of post-market information; however, to date, there is no record of Health Canada ordering the production or submission of information under Vanessa’s Law.

Finally, it is unclear how post-market trial results are assessed to support removal of conditions. As described above (see Table 5), the statement of withdrawal included in the QN varies significantly in terms of describing the evidence threshold that must be demonstrated. In some cases, the QN states indications can be withdrawn if confirmatory trials are ‘unsuccessful’, while others state that the trial must demonstrate an OS benefit. As a result, there does not appear to be a standard threshold for post-market confirmatory trial results, and Health Canada does not publish decisions describing how post-market evidence was assessed. Under the PRCI, Health Canada has started proactively releasing the clinical information packages submitted to support regulatory decisions, including transfer from NOC/c to NOC. Such packages including overwhelming amounts of clinical information, such that it is not reasonable to assume that patients, clinicians, or other stakeholders would be capable to reviewing them.

It is currently unclear whether post-market commitments are tailored to address uncertainties that existed at the time of authorization and how the results of post-market trials are assessed, but there are several measures that could be implemented to ensure post-market trials are suitably designed and evaluated. For example, developing and maintaining an inventory of acceptable surrogate endpoints would provide certainty to manufacturers when designing pre- and post-market trials, and could improve consistency in regulatory decision-making.^96^ Regulators could also require post-market trials to be randomized, except in cases where such an approach would be overly burdensome or where observational trials are deemed sufficient to address the uncertainties.^97^ Conducting post-market randomized trials can create recruitment challenges because patients are often reluctant to participate in trials and risk receiving a placebo or standard of care when they can receive the drug outside of the trial. Increased reliance on real-world data for post-market evidence generation could address this concern. Current limitations of real-world data generation and use in Canada prevent this approach from being used widely, absent significant legal and infrastructural updates. Increased transparency about post-market trials, specifically the regulatory assessment of submitted results, would go a long way to increasing accountability and clarifying how post-market trial data are assessed. Ideally, clinical thresholds sufficient for removal of conditions should be determined and published at the time of authorization.

### VI.E. Enhancing Enforcement and Compliance

Domestic and international experiences with drugs authorized under life-cycle regulatory pathways demonstrate that enforcement and compliance are often lacking.^98^ Failure to submit data required may put patients at unnecessary risk and complicates clinical decision-making. Concerns exist about both compliance with and enforcement of requirements to submit post-market confirmatory trials. Absent voluntary compliance, regulators may be limited in their ability to respond to drugs that fail to demonstrate clinical benefit in confirmatory trials. Our review of drugs authorized under the NOC/c policy demonstrates that post-market confirmatory trials are not always completed or submitted in a timely fashion. As of May 2022, there were 25 drugs that had been authorized under the NOC/c policy and remained authorized under the NOC/c policy for at least four years without satisfying the post-market conditions. Additionally, approximately one-quarter of drugs authorized under the NOC/c policy with active conditions in place had clinical trials listed as complete on the clinicaltrials.gov database (Table 6). We also identified five authorizations as converted to standard authorization in the United States but still authorized under the NOC/c policy in Canada (Table 7), with no clear reason for the delay in regulatory response. It is possible that the results have been received and/or reviewed by Health Canada, but the PAAT has not been updated. Health Canada relies on drug manufacturers to provide estimated timelines for submitting required clinical trial reports and other evidence, but in many cases, the QNs do not specify a timeline.

**Table 6:** Drugs Authorized under NOC/c Policy with Active Conditions and Confirmatory Trials Listed as Complete

Drug	NOC/c Date	Confirmatory trial(s) identifier & status^*^
Brigatinib	7/26/2018	(1) ALTA-1 L: Completed, results posted 8/20/2021
Blinatumomab	12/19/2019	(1) E910: Active, not recruiting (2) AALL1331: Active, not recruiting (3) MT103-203: Completed, results posted 2/12/2015
Ponatinib	4/2/2015	(1) AP24534-10-20: Completed, results posted 1/29/2020 (2) AP24534-14-203: Active, not recruiting
Enasidenib	2/6/2019	(1) AG221-AML-004: Recruiting (2) CC90007-CP-004: Recruiting (3) CC90007-CP-003: Completed
Durvalumab	11/3/2017	(1) D419BC00001 (DANUBE): Active, note recruiting (2) CD-ON-MEDI4736-1108: Completed
Sebelipase alfa	12/15/2017	(1) LALD Registry: Recruiting (2) LAL-CL04: Completed, results posted 5/9/2016 (3) LAL-CL02: Completed, results posted 4/18/2016 (4) LAL-CL03: Completed, results posted 4/18/2016 (5) LAL-CL06: Completed, results posted 1/15/2019 (6) LAL-CL08: Terminated, results posted
Pembrolizumab	9/21/2018	(1) KEYNOTE-170: Completed, results posted 6/24/2020 (2) KN204: Active, not recruiting (3) KEYNOTE-051: Recruiting
Cemiplimab	4/10/2019	(1) R2810-ONC-1540: Recruiting (2) R2810-ONC-1423: Completed
Nivolumab	11/10/2017	(1) CA209067: Active, not recruiting, results first posted 9/26/2017 (2) CA209812: Active, not recruiting (3) CA209205: Active, not recruiting, results posted 12/11/2018 (4) CA209744: Recruiting
Nivolumab	3/23/2018	(1) CA209459: Active, not recruiting, results posted 6/26/2020
Entrectinib	2/10/2020	(1) ALKA: Unknown (2) STARTRK-1: Completed (3) STARTRK-2: Recruiting
Asfotase alfa	8/14/2015	(1) ENB-006-09: Completed, results posted 7/26/2017 (2) ENB-008-10: Completed, results posted 7/26/2017 (3) ENB-010-10: Completed, results posted 2/26/2018 (4) ENB-009-10: Completed, results posted 9/18/2017 (5) ENB-002-08: Completed, results posted 9/30/2011 (6) ENB-003-08: Completed, results posted 11/17/2017 (7) ENB-0040: Unknown
Remdesivir	7/27/2020	(1) CO-US-540-5776: Unknown (2) GS-US-540-5773: Completed, results posted 12/31/2020 (3) GS-US-540-5774: Completed, results posted 1/26/2021 (4) CO-US-540-5758: Unknown
Ceritinib	3/27/2015	(1) CLDK378A2303: Active, not recruiting, results posted 7/27/2017 (2) CLDK378BX2101: Completed, results posted 8/11/2014 (3) CLDK378X1101: Completed

**Table 7:** Drugs Authorized under NOC/c Policy and AA, with Active Conditions in Canada but Converted to Standard Approval in the United States

Drug	NOC/c date	Indication	NOC/c status (8/30/2021)	US FDA AA Date	AA status (8/30/2021)
1. Pembrolizumab	9/21/2018	Relapsed or refractory primary mediastinal B-cell lymphoma	Active	6/13/2018	Converted 10/14/2020
2. Pembrolizumab	2/5/2021	Refractory or relapsed classical Hodgkin lymphoma	Active	3/14/2017	Converted 10/14/2020
3. Ceritinib	3/27/2015	Anaplastic lymphoma kinase positive (ALK+) NSCLC (second line)	Active	4/29/2014	Converted 5/26/2017
4. Brigatinib	7/26/2018	ALK+ NSCLC (second line)	Active	4/28/2017	Converted 5/22/2020
5. Blinatumomab	4/28/2017	Pediatric patients with Ph- relapsed or refractory B-cell precursor ALL	Active	9/1/2016	Converted 7/11/2017
6. Ponatinib hydrochloride	4/2/2015	Chronic myelogenous leukemia (CML) or Ph + ALL	Active	12/14/2012	Converted 11/28/2016

Health Canada has a suite of escalating enforcement tools available if confirmatory trials fail to confirm clinical benefit or identify new safety risks. To date, Health Canada has largely relied on voluntary self-enforcement to withdraw indications where post-market confirmatory trials fail to confirm clinical efficacy. Of all drug-indications authorized under the NOC/c policy to date, three have been suspended. Health Canada used its regulatory power under the Food and Drug Regulations to suspend bicalutamide for prostate cancer based on evidence of increased mortality.^99^ Celecoxib for familial adenomatous polyposis was also suspended after an interim analysis of the confirmatory trial showed a statistically significant increase in the risk of heart attack, stroke, and cardiovascular death.^100^ Bevacizumab for breast cancer is also listed as suspended; however, no official justification from Health Canada could be identified. Additionally, 15 drug-indications authorized under the NOC/c policy have been withdrawn or discontinued by the manufacturer. When this happens, no justification for the withdrawal or discontinuation is provided.

In other cases, Health Canada has allowed drugs to stay on the market following the failure of post-market clinical trials, instead limiting access to patients already taking the drug, publishing safety advisories, or changing the post-market requirements. Two instances of limiting patient access were identified. Gefitinib, authorized under the NOC/c policy in 2003 for non-small cell lung cancer, failed to demonstrate an improvement in OS in the confirmatory trial. Rather than suspend the authorization, Health Canada allowed gefitinib to remain on market, but indicated that no new patients should initiate treatment. Additionally, patients were required to join a registry.^101^ In another case, patient access was restricted at the time of authorization. Ponatinib was authorized under the NOC/c policy for chronic myeloid leukemia and acute lymphoblastic leukemia on the condition that it would only be prescribed by a physician certified with the Iclusig Controlled Distribution Program.^102^

Several instances of Health Canada amending the authorized indication to include a caveat following completion of confirmatory trials were identified. For example, three aromatase inhibitors were approved under the NOC/c pathway between 2004 and 2006 for the adjuvant treatment of postmenopausal women with breast cancer based on disease-free survival. When the conditions were removed, there was no significant difference in OS compared to standard of care (tamoxifen) or placebo. Instead of withdrawing the indication or requesting new trials to resolve evidentiary uncertainties, these drugs had caveats attached to their indications in the product monograph stating that there is no demonstrated OS benefit.^103^

Concerns also exist with respect to Health Canada’s response to foreign regulatory decisions. Over the last year, three drugs authorized in Canada under the NOC/c policy have either been voluntarily withdrawn or recommended for withdrawal in the United States due to clinical trials that failed to confirm benefit (Table 8). Despite a requirement in the NOC/c Guidance Document for drug manufacturers to notify Health Canada of regulatory action in another jurisdiction, it is unclear whether these requirements have been honored, and if so, why Health Canada has not acted. Inconsistent regulatory decision-making is a barrier to health technology life-cycle regulation.

**Table 8:** Drugs Authorized under NOC/c Policy that have been Withdrawn or Recommended to be Withdrawn in the United States

Drug	NOC/c Date	Indication	NOC/c status (8/30/2021)	US FDA AA Date	AA status (8/30/2021)
Durvalumab for injection	11/3/2017	Locally advanced or metastatic urothelial carcinoma (second line)	Active	5/1/2017	Voluntarily withdrawn
Nivolumab	3/23/2018	Advanced or metastatic hepatocellular carcinoma (second line)	Active	9/22/2017	Voluntarily withdrawn
Atezolizumab	4/12/2017	Urothelial carcinoma (second line)	Active	5/18/2016	Voluntarily withdrawn
Gefitinib	12/17/2003	Locally advanced or metastatic NSCLC (third line)	Transferred	5/5/2003	Withdrawn 4/25/2012

Timely completion of post-market trials is an oft-cited concern associated with life-cycle regulatory authorization paradigms.^104^ Indeed, Health Canada recognized this concern as early as 2000 in an internal review.^105^ Many suggestions have been made to ameliorate the ongoing challenge with enforcing post-market confirmatory trials: stronger penalties; clear, publicized timelines; automatic expiration of authorization without sufficient trial progress; mandatory publication of clinical trial results; and requiring confirmatory trials to be underway at the time of authorization.^106^

In addition to policy design features to encourage and increase compliance, enforcement of post-market confirmatory trials that fail to demonstrate predetermined efficacy thresholds is also required. There are several examples in Canada and internationally of post-market trials that failed to demonstrate or confirm clinical benefit, yet languished on market for months or years, were granted a second chance to perform an additional clinical trial, or had the conditions removed and were transferred to a full authorization. For example, olaratumab was authorized under the NOC/c policy in 2017 for soft tissue sarcoma. In 2019, the results of the required post-market trial were submitted to Health Canada, which did not confirm the clinical benefit of the drug. Health Canada issued a Dear Health Care Professional Letter to notify physicians that new patients should not start olaratumab, but the indication was not officially withdrawn for almost two years.^107^ It is clear that new approaches for managing underperforming post-market trials are necessary.

Currently, Health Canada relies on drug manufacturers to voluntarily withdraw authorized indications, requiring negotiations between Health Canada and the manufacturer, during which time, new patients may start or continue treatment with the drug, forgoing other safer or more effective options, all without knowledge of the results of the confirmatory trial. This may be exacerbated by the lack of clear and consistent criteria or threshold that must be met to remove conditions, as described above. Health Canada’s process to assess post-market clinical trials remains opaque. A standardized process should be developed for managing post-market confirmatory trials that fail to confirm clinical benefit, including necessary appeals for parties that disagree with the interpretation of the results. Depending on the circumstances, it may be appropriate to ensure that no new patients start on the drug, existing patients who are benefitting from treatment are transferred to a special access program, or that treatment is withdrawn entirely, in a safe and controlled manner. Most importantly, because of the inherent difficulties with withdrawing regulatory authorization, even when it is conditional, trends toward normalizing ‘drug exceptionalism’ should be resisted so that conditional authorizations remain the exception, and not the rule.

## VII. LIMITATIONS TO OUR ANALYSIS

Our study has some limitations. Information on NOC/cs granted between 1998 and 2004 was not available through the Wayback Machine, so this analysis likely underestimates the total number of drugs authorized under the NOC/c policy, particularly for this period. In addition, many Health Canada websites and databases are not up to date. There is a time lag between when drugs are authorized and when RDSs and SBDs are published, so these documents are not typically available for up to two years after authorization. There was significant variability in the amount of information available for each drug/indication, depending on what documents were publicly available. In addition, there were often discrepancies in the information provided between sources. We did not study the characteristics of pivotal trials in drugs authorized under the standard regulatory authorization process, so we cannot state conclusively that our findings are unique to the NOC/c policy. For the uncertainty assessment, we only labelled uncertainties where Health Canada documents specifically mentioned them. We did not assess the pivotal trial(s) for uncertainties. As uncertainties are not assessed in a comprehensive or standardized manner, it is possible that our findings are not representative of the actual uncertainties contemplated by Health Canada during the review process.

## VIII. CONCLUSION

As Health Canada and global health regulators increasingly move toward life-cycle regulatory approaches, experience to date with conditional regulatory pathways provides useful insights for specific policy approaches and tools that can be adopted to mitigate challenges and gaps identified under existing conditional approaches. Health Canada is currently in the process of designing and implementing two new regulatory approaches: the broad use of terms and conditions on therapeutic product authorizations and the regulatory sandbox for advanced therapeutic products. These approaches provide a more flexible, context-based regulatory approach, which blurs the line between pre- and post-market oversight.

We collected and analyzed the largest sample of NOC/c authorizations published to date and analyzed a sample of 148 drugs authorized between 1998 and 2021, including characteristics about the pre- and post-market clinical trials. We found that most NOC/c authorizations are based on one, single-arm clinical trial using a surrogate endpoint. Post-market trials are more likely to be randomized, Phase III trials but still rely heavily on surrogate endpoints. Many of our findings align with earlier analyses of the NOC/c policy, as well as international experience with AA in the United States and CMA in the European Union.

Our analysis of the NOC/c policy led to recommendations for policy design of life-cycle regulatory approaches. First, we recommend increased decision-making transparency throughout the regulatory process, starting with pre-market evidence review, to selecting and designing post-market conditions and removing conditions to provide greater accountability and to promote informed decision-making by stakeholders. Second, we recommend developing comprehensive eligibility criteria and guidance for selecting appropriate health technologies that benefit from more flexible regulatory decision-making to ensure that the benefits of earlier access outweigh the risks of access based on earlier evidence. Third, we recommend modernizing pre-market evidence requirements to clearly permit flexibility in recognition of the limits of the ‘two RCT’ approach, coupled with proactive transparency and standardization to promote accountability and informed decision-making. This recommendation reflects existing exercise of flexibility by regulators in interpreting evidence requirements, but no clear framework exists for how regulators determine when to exercise of flexibility and its extent. Additionally, this should include developing a list or database of acceptable surrogate markers. Fourth, we recommend Health Canada adopt a more active role in guiding the design of post-market trials to ensure that they can address the pre-market uncertainties. Again, transparency here is critical to communicate effectively to stakeholders. Finally, upon consideration of the challenges experienced with completing post-market conditions, we recommend adopting policy tools used and proposed in other jurisdictions, such as automatic expiry, stronger penalties, and ongoing disclosure of the status of post-market trials.

Enforcement of post-market trials that fail to confirm benefit is a significant concern that must be addressed to preserve the ‘social compromise’ of conditional regulatory pathways.^108^ Rather than relying on manufacturers to voluntarily withdraw drugs that fail to confirm clinical benefit, clear decision frameworks and standardized procedures should be established at the time of authorization to limit disputes, including how to protect patients during the withdrawal process. The proposed terms and conditions amendments clearly grant Health Canada enforcement authority, representing an improvement over the voluntary approach under the NOC/c policy. However, such an authority must be used to foster public trust in life-cycle regulatory approaches. Common to these recommendations is a radical increase in transparency throughout the regulatory life cycle of a product, which we believe is critical to empower stakeholders, such as patients, physicians, and reimbursement decision-makers, to make informed decisions, and promote regulatory consistency and accountability.

## Conflict of interest

The authors declare that the research was conducted in the absence of any commercial or financial relationships that could be construed as a potential conflict of interest.

## Funding

The Canadian Network for Learning Healthcare Systems and Cost-effective ‘Omics Innovation is funded by Genome British Columbia/Genome Canada (G05CHS).

## Supplementary Material

Appendix_1_lsad008

## Footnotes

1 See eg US: Sanket S. Dhruva & Rita F. Redberg, *Accelerated approval and possible withdrawal of midodrine*, 304 JAMA 2172–173 (2010).; Bishal Gyawali & Aaron S. Kesselheim, *Reinforcing the social compromise of accelerated approval*, 15 Nat Rev Clin Oncol 596–97 (2018).; Bishal Gyawali, Spencer Phillips Hey & Aaron S. Kesselheim, *Assessment of the Clinical Benefit of Cancer Drugs Receiving Accelerated Approval*, 179 JAMA Intern Med 906 (2019).; Julia A. Beaver & Richard Pazdur, *‘Dangling’ Accelerated Approvals in Oncology*, 384 N Engl J Med e68 (2021).; Jonathan J Darrow, *Few new drugs deserve expedited regulatory treatment*, 27 JMCP 685–88 (2021).; Julia A. Beaver et al., *A 25-Year Experience of US Food and Drug Administration Accelerated Approval of Malignant Hematology and Oncology Drugs and Biologics: A Review*, 4 JAMA Oncol 849 (2018).; Huseyin Naci, Katelyn R. Smalley & Aaron S. Kesselheim, *Characteristics of Preapproval and Postapproval Studies for Drugs Granted Accelerated Approval by the US Food and Drug Administration*, 318 JAMA 626–36 (2017).; Anna Kaltenboeck, Amanda Mehlman & Steven D. Pearson, *Strengthening the Accelerated Approval Pathway: An Analysis of Potential Policy Reforms and their Impact on Uncertainty, Access, Innovation, and Costs [White Paper]*, (2021), https://icer.org/wp-content/uploads/2021/04/Strengthening-the-Accelerated-Approval-Pathway-_-ICER-White-Paper-_-April-2021.pdf (accessed July 7, 2022).; Bishal Gyawali, Benjamin N Rome & Aaron S Kesselheim, *Regulatory and clinical consequences of negative confirmatory trials of accelerated approval cancer drugs: retrospective observational study*, BMJ n1959 (2021).; For European Union analyses, see eg J. Hoekman et al., *Use of the conditional marketing authorization pathway for oncology medicines in Europe*, 98 Clin Pharmacol Ther 534–41 (2015).; European Medicines Agency, *Conditional Marketing Authorisation: Report on ten years of experience at the European Medicines Agency*, (2017), https://www.ema.europa.eu/en/documents/report/conditional-marketing-authorisation-report-ten-years-experience-european-medicines-agency_en.pdf (accessed July 4, 2022).; Jarno Hoekman et al., *Characteristics and follow-up of postmarketing studies of conditionally authorized medicines in the EU*, 82 Br J Clin Pharmacol 213–26 (2016).; Jarno Hoekman & Wouter Boon, *Changing standards for drug approval: A longitudinal analysis of conditional marketing authorisation in the European Union*, 222 Soc Sci Med 76–83 (2019).

2 Joel Lexchin, *Notice of compliance with conditions: a policy in limbo*, 2 Healthc Policy 114–22 (2007).; Joel Lexchin, *Publication of confirmatory studies required by Health Canada for drugs approved under a Notice of Compliance with conditions: a cohort study*, 5 CMAJ Open E295–E300 (2017).; Joel Lexchin, *Quality of evidence considered by Health Canada in granting full market authorisation to new drugs with a conditional approval: a retrospective cohort study*, 8 BMJ Open e020377 (2018).; Joel Lexchin, *Health Canada’s use of accelerated review pathways and therapeutic innovation, 1995–2016: cross-sectional analysis*, 8 BMJ Open e023605 (2018).; Michael R. Law, *The characteristics and fulfillment of conditional prescription drug approvals in Canada*, 116 Health Policy 154–61 (2014).; Joel Lexchin, *Post-market safety warnings for drugs approved in Canada under the Notice of Compliance with conditions policy*, 79 Br J Clin Pharmacol 847–59 (2015).; S. K. Andersen et al., *Conditional approval of cancer drugs in Canada: accountability and impact on public funding*, 26 Curr Oncol e100–e105 (2019).

3 Joel Lexchin, *Notice of Compliance With Conditions: A Policy In Limbo*, 2 Healthc Policy 114–22 (2007).

4 Health Canada Government of Canada, *Guidance Document: Notice of Compliance with Conditions (NOC/c)*, (2016), (accessed July 4, 2022).

5
*Food and Drugs Act*, RSC, 1985, c. F-27.

6
*Food and Drug Regulations,* CRC c 180.

7 Health Canada Government of Canada, *Guidance Document: Notice of Compliance with Conditions (NOC/c)*, (2016), https://www.canada.ca/content/dam/hc-sc/migration/hc-sc/dhp-mps/alt_formats/pdf/prodpharma/applic-demande/guide-ld/compli-conform/noccg_accd-eng.pdf (accessed July 4, 2022).

8 Health Canada Government of Canada, *Guidance Document: Notice of Compliance with Conditions (NOC/c)*, (2016), https://www.canada.ca/content/dam/hc-sc/migration/hc-sc/dhp-mps/alt_formats/pdf/prodpharma/applic-demande/guide-ld/compli-conform/noccg_accd-eng.pdf (accessed July 4, 2022).

9 Health Canada Government of Canada, *Guidance Document: Notice of Compliance with Conditions (NOC/c)*, (2016), https://www.canada.ca/content/dam/hc-sc/migration/hc-sc/dhp-mps/alt_formats/pdf/prodpharma/applic-demande/guide-ld/compli-conform/noccg_accd-eng.pdf (accessed July 4, 2022).

10 Health Canada Government of Canada, *Guidance Document: Notice of Compliance with Conditions (NOC/c)*, (2016), https://www.canada.ca/content/dam/hc-sc/migration/hc-sc/dhp-mps/alt_formats/pdf/prodpharma/applic-demande/guide-ld/compli-conform/noccg_accd-eng.pdf (accessed July 4, 2022).

11 Health Canada Government of Canada, *Guidance Document: Notice of Compliance with Conditions (NOC/c)*, (2016), https://www.canada.ca/content/dam/hc-sc/migration/hc-sc/dhp-mps/alt_formats/pdf/prodpharma/applic-demande/guide-ld/compli-conform/noccg_accd-eng.pdf (accessed July 4, 2022), at 2.3, 2.5, 2.6.

12
*Food and Drug Regulations*, CRC c 180, s C.01.016; Health Canada Government of Canada, *Guidance Document: Notice of Compliance with Conditions (NOC/c)*, (2016), https://www.canada.ca/content/dam/hc-sc/migration/hc-sc/dhp-mps/alt_formats/pdf/prodpharma/applic-demande/guide-ld/compli-conform/noccg_accd-eng.pdf (accessed July 4, 2022).

13 Health Canada Government of Canada, *Guidance Document: Notice of Compliance with Conditions (NOC/c)*, (2016), https://www.canada.ca/content/dam/hc-sc/migration/hc-sc/dhp-mps/alt_formats/pdf/prodpharma/applic-demande/guide-ld/compli-conform/noccg_accd-eng.pdf (accessed July 4, 2022).

14
*Food and Drug Regulations,* CRC c 180, ss C.01.018–019.

15 Health Canada Government of Canada, *Guidance Document: Notice of Compliance with Conditions (NOC/c)*, (2016), https://www.canada.ca/content/dam/hc-sc/migration/hc-sc/dhp-mps/alt_formats/pdf/prodpharma/applic-demande/guide-ld/compli-conform/noccg_accd-eng.pdf (accessed July 4, 2022).

16 Health Canada Government of Canada, *Regulatory Requirements for Advertising*, (2015), https://www.canada.ca/en/health-canada/services/drugs-health-products/regulatory-requirements-advertising.html (accessed July 4, 2022).

17 Health Canada Government of Canada, *Health Product InfoWatch*, (2020), https://www.canada.ca/en/health-canada/services/drugs-health-products/medeffect-canada/health-product-infowatch.html (accessed July 30, 2020).

18 Health Canada Government of Canada, *Guidance Document: Notice of Compliance with Conditions (NOC/c)*, (2016), https://www.canada.ca/content/dam/hc-sc/migration/hc-sc/dhp-mps/alt_formats/pdf/prodpharma/applic-demande/guide-ld/compli-conform/noccg_accd-eng.pdf (accessed July 4, 2022).

19 Health Canada Government of Canada, *Guidance Document: Notice of Compliance with Conditions (NOC/c)*, (2016), https://www.canada.ca/content/dam/hc-sc/migration/hc-sc/dhp-mps/alt_formats/pdf/prodpharma/applic-demande/guide-ld/compli-conform/noccg_accd-eng.pdf (accessed July 4, 2022).; Food and Drug Regulations, CRC c 180

20 Matthew Herder, *Pharmaceutical Drugs of Uncertain Value, Lifecycle Regulation at the US Food and Drug Administration, and Institutional Incumbency*, 97 Milbank Quarterly 820–857 (2019).

21 Lawrence Liberti et al, *Accelerating access to new medicines: Current Status of Facilitated Regulatory Pathways Used by Emerging Regulatory Authorities, 37:3*Journal of Public Health Policy 315–333 (2016).

22 Health Canada Government of Canada, *Guidance for Industry - Priority Review of Drug Submissions*, (2005), https://www.canada.ca/en/health-canada/services/drugs-health-products/drug-products/applications-submissions/guidance-documents/priority-review/drug-submissions.html (accessed July 4, 2022), at 1.4.

23 Health Canada Government of Canada, *Guidance for Industry - Priority Review of Drug Submissions*, (2005), https://www.canada.ca/en/health-canada/services/drugs-health-products/drug-products/applications-submissions/guidance-documents/priority-review/drug-submissions.html (accessed July 4, 2022), at 1.4.

24 Health Canada Government of Canada, *Priority Review of Drug Submissions (Therapeutic Products)*, https://www.canada.ca/content/dam/hc-sc/migration/hc-sc/dhp-mps/alt_formats/hpfb-dgpsa/pdf/prodpharma/prfs_tpfd-eng.pdf (accessed July 4, 2022).

25 Health Canada Government of Canada, *Guidance Document: Notice of Compliance with Conditions (NOC/c)*, (2016), https://www.canada.ca/en/health-canada/services/drugs-health-products/drug-products/applications-submissions/guidance-documents/notice-compliance-conditions.html (accessed July 4, 2022).

26 Health Canada Government of Canada, *Consultation on Proposed Agile Regulations and Guidance for Licensing Drugs and Medical Devices* (2023), https://www.canada.ca/en/health-canada/programs/consultation-proposed-agile-regulations-guidance-licensing-drugs-medical-devices.html (accessed Jan. 27, 2023).

27
*Food and Drugs Act, ss.* 3.2, 21.7, 21.92, 30(1)(b.3), 30(1.2)(b).

28 Health Canada Government of Canada, *ARCHIVED Interim Order Respecting the Importation, Sale and Advertising of Drugs for Use in Relation to COVID-19*, (2021), https://www.canada.ca/en/health-canada/services/drugs-health-products/covid19-industry/drugs-vaccines-treatments/interim-order-import-sale-advertising-drugs.html (accessed Sep. 16, 2021).

29 Public Works and Government Services Canada Government of Canada, *Canada Gazette, Part 2, Volume 155, Number 7: Regulations Amending the Food and Drug Regulations (Interim Order Respecting the Importation, Sale and Advertising of Drugs for Use in Relation to COVID-19)*, (2021), https://canadagazette.gc.ca/rp-pr/p2/2021/2021-03-31/html/sor-dors45-eng.html (accessed July 4, 2022).

30 Government of Canada, *Canada Gazette, Part I, Volume 156, Number 51: Regulations Amending Certain Regulations Made Under the Food and Drugs Act (Agile Licensing)* (2022), https://canadagazette.gc.ca/rp-pr/p1/2022/2022-12-17/html/reg1-eng.html (accessed Jan. 28, 2023).

31 Joel Lexchin, *Publication of Confirmatory Studies Required by Health Canada for Drugs Approved Under a Notice of Compliance with Conditions: A Cohort Study*, 5 CMAJ Open E295–E300 (2017).

32 Joel Lexchin, *Quality of Evidence Considered by Health Canada in Granting Full Market Authorisation to New Drugs With a Conditional Approval: A Retrospective Cohort Study*, 8 BMJ Open e020377 (2018).

33 Joel Lexchin, *Health Canada’s Use of Accelerated Review Pathways And Therapeutic Innovation, 1995–2016: cross-sectional analysis*, 8 BMJ Open e023605 (2018).

34 Joel Lexchin, *Post-Market Safety Warnings For Drugs Approved in Canada Under the Notice of Compliance with Conditions Policy*, 79 Br J Clin Pharmacol 847–59 (2015).

35 Michael R. Law, *The Characteristics and Fulfillment of Conditional Prescription Drug Approvals in Canada*, 116 Health Policy 154–61 (2014).

36 S. K. Andersen et al., *Conditional Approval of Cancer Drugs in Canada: Accountability and Impact on Public Funding*, 26 Curr Oncol e100–e105 (2019).

37
Federal Food, Drug & Cosmetic Act, 21 USC § 506(c).

38 21 CFR §§ 314.500, 601.40.; U.S. Department of Health and Human Services et al., *Guidance for Industry Expedited Programs for Serious Conditions – Drugs and Biologics*, (2014), https://www.fda.gov/media/86377/download (accessed July 4, 2022). The guidance document clarifies that even though the eligibility states the drug must provide a benefit over existing therapies, FDA can also consider the lack of alternative treatments. This aligns with international interpretations of ‘unmet medical need’ which includes both a therapeutic area with no available therapies, and providing an improvement over existing therapies. For further discussion on this distinction, see Melanie McPhail, Emma Weiss & Tania Bubela, *Conditional Drug Approval as a Path to Market For Oncology Drugs in Canada: Challenges and Recommendations for Assessing Eligibility and Regulatory Responsiveness*, 8 Frontiers in medicine (2022).

39
Federal Food, Drug & Cosmetic Act, 21 USC § 505(d).

40 It is worth noting that surrogate endpoints are also accepted under the traditional approval process. In such cases, post-marketing confirmatory studies may be requested, and often are, but are not required.

41
Federal Food, Drug & Cosmetic Act, 21 USC § 506(c).; U.S. Food and Drug Administration, *Accelerated Approval*, FDA (2018), https://www.fda.gov/patients/fast-track-breakthrough-therapy-accelerated-approval-priority-review/accelerated-approval (accessed July 6, 2022).

42 U.S. Department of Health and Human Services et al., *Guidance for Industry Expedited Programs for Serious Conditions – Drugs and Biologics*, (2014), https://www.fda.gov/media/86377/download (accessed July 4, 2022).

43 Sanket S. Dhruva & Rita F. Redberg, *Accelerated approval and possible withdrawal of midodrine*, 304 JAMA 2172–173 (2010).; Bishal Gyawali & Aaron S. Kesselheim, *Reinforcing the Social Compromise of Accelerated Approval*, 15 Nat Rev Clin Oncol 596–97 (2018).; Bishal Gyawali, Spencer Phillips Hey & Aaron S. Kesselheim, *Assessment of the Clinical Benefit of Cancer Drugs Receiving Accelerated Approval*, 179 JAMA Intern Med 906 (2019).; Julia A. Beaver & Richard Pazdur, *‘Dangling’ Accelerated Approvals in Oncology*, 384 N Engl J Med e68 (2021).; Jonathan J Darrow, *Few New Drugs Deserve Expedited Regulatory Treatment*, 27 JMCP 685–88 (2021).; Julia A. Beaver et al., *A 25-Year Experience of US Food and Drug Administration Accelerated Approval of Malignant Hematology and Oncology Drugs and Biologics: A Review*, 4 JAMA Oncol 849 (2018).; Huseyin Naci, Katelyn R. Smalley & Aaron S. Kesselheim, *Characteristics of Preapproval and Postapproval Studies for Drugs Granted Accelerated Approval by the US Food and Drug Administration*, 318 JAMA 626–36 (2017).; Anna Kaltenboeck, Amanda Mehlman & Steven D. Pearson, *Strengthening the Accelerated Approval Pathway: An Analysis of Potential Policy Reforms and their Impact on Uncertainty, Access, Innovation, and Costs [White Paper.]*, (2021), https://icer.org/wp-content/uploads/2021/04/Strengthening-the-Accelerated-Approval-Pathway-_-ICER-White-Paper-_-April-2021.pdf (accessed July 7, 2022).; Bishal Gyawali, Benjamin N Rome & Aaron S Kesselheim, *Regulatory and Clinical Consequences of Negative Confirmatory Trials of Accelerated Approval Cancer Drugs: Retrospective Observational Study*, BMJ n1959 (2021).

44 Gautam U. Mehta, R. Angelo de Claro and Richard Pazdur, *Accelerated Approval is Not Conditional Approval: Insights From International Expedited Approval Programs*, 8 JAMA Oncol 335–336.

45 Anna Kaltenboeck, Amanda Mehlman & Steven D. Pearson, *Strengthening the Accelerated Approval Pathway: An Analysis of Potential Policy Reforms and their Impact on Uncertainty, Access, Innovation, and Costs [White Paper.]*, (2021), https://icer.org/wp-content/uploads/2021/04/Strengthening-the-Accelerated-Approval-Pathway-_-ICER-White-Paper-_-April-2021.pdf (accessed July 7, 2022).; Huseyin Naci, Katelyn R. Smalley & Aaron S. Kesselheim, *Characteristics of Preapproval and Postapproval Studies for Drugs Granted Accelerated Approval by the US Food and Drug Administration*, 318 JAMA 626–36 (2017); Elisabeth Mahase, *FDA Allows Drugs Without Proven Clinical Benefit to Languish For Years on Accelerated Pathway*, 374 BMJ n1898 (2021); Erin A. Ferries, William K. Fleming & William H. Shrank, *FDA Expedited Approval and Implications For Rational Formulary And Health Plan Design*, 27 J Manag Care Spec Pharm 682–84 (2021); Aaron S Kesselheim et al., *Trends in Utilization of FDA Expedited Drug Development And Approval Programs, 1987–2014: Cohort Study*, BMJ h4633 (2015).

46 21 CFR § 314;Herder, *supra* note 20.

47 U.S. Food and Drug Administration, *April 27–29, 2021: Meeting of the Oncologic Drugs Advisory Committee Meeting Announcement - 04/27/2021–04/29/2021*, FDA (2021), https://www.fda.gov/advisory-committees/advisory-committee-calendar/april-27-29-2021-meeting-oncologic-drugs-advisory-committee-meeting-announcement-04272021-04292021 (accessed July 6, 2022).

48 Food and Drug Omnibus Reform Act, H.R. 2617, 117th Congress (2021–2022).

49 U.S. Department of Health and Human Services et al., *Guidance for Industry Expedited Programs for Serious Conditions – Drugs and Biologics*, (2014), https://www.fda.gov/media/86377/download (accessed July 4, 2022).

50 European Union, *COMMISSION REGULATION (EC) No 507/2006 of 29 March 2006 on the Conditional Marketing Authorisation for Medicinal Products for Human Use Falling Within The Scope of Regulation (EC) No 726/2004 of the European Parliament and of the Council*, L 92/6 Official Journal of the European Union (2006), https://eur-lex.europa.eu/legal-content/EN/TXT/PDF/?uri=CELEX:32006R0507&from=IT (accessed July 6, 2022).; European Medicines Agency & Committee for Medicinal Products for Human Use, *Guideline on the Scientific Application and the Practical Arrangements Necessary to Implement Commission Regulation (EC) No 507/2006 on the Conditional Marketing Authorisation For Medicinal Products for Human Use Falling Within The Scope of Regulation (EC) No 726/2004*, (2016), https://www.ema.europa.eu/en/documents/scientific-guideline/guideline-scientific-application-practical-arrangements-necessary-implement-commission-regulation-ec/2006-conditional-marketing-authorisation-medicinal-products-human-use-falling_en.pdf (last visited July 6, 2022).

51 Committee for Medicinal Products for Human Use European Medicines Agency, *Guideline on Procedures for the Granting of a Marketing Authorisation under Exceptional Circumstances, Pursuant to Article 14(8) of Regulation (EC) No 726/2004*, (2005), https://www.ema.europa.eu/en/documents/regulatory-procedural-guideline/guideline-procedures-granting-marketing-authorisation-under-exceptional-circumstances-pursuant/2004_en.pdf (accessed July 6, 2022).

52 J. Hoekman et al., *Use of the Conditional Marketing Authorization Pathway for Oncology Medicines in Europe*, 98 Clin Pharmacol Ther 534–41 (2015).; European Medicines Agency, *Conditional Marketing Authorisation: Report on Ten Years of Experience at the European Medicines Agency*, (2017), https://www.ema.europa.eu/en/documents/report/conditional-marketing-authorisation-report-ten-years-experience-european-medicines-agency_en.pdf (accessed July 4, 2022).; Jarno Hoekman et al., *Characteristics and Follow-up of Postmarketing Studies of Conditionally Authorized Medicines in the EU*, 82 Br J Clin Pharmacol 213–26 (2016).; Jarno Hoekman & Wouter Boon, *Changing Standards for Drug Approval: A Longitudinal Analysis of Conditional Marketing Authorisation in the European Union*, 222 Soc Sci Med 76–83 (2019).

53 J. Hoekman et al., *Use of the Conditional Marketing Authorization Pathway for Oncology Medicines in Europe*, 98 Clin Pharmacol Ther 534–41 (2015).

54 Health Canada Government of Canada, *Notice of Compliance with conditions (NOC/c)*, (2021), https://www.canada.ca/en/health-canada/services/drugs-health-products/drug-products/notice-compliance/conditions.html (accessed July 6, 2022).

55 Health Canada Government of Canada, *Notice of Compliance Search*, (2021), https://health-products.canada.ca/noc-ac/index-eng.jsp (accessed July 6, 2022).

56 Health Canada Government of Canada, *Drug Product Database Online Query*, (2012), https://health-products.canada.ca/dpd-bdpp/index-eng.jsp (accessed July 6, 2022).

57 Health Canada Government of Canada, *Drug and Health Product Submissions Under Review (SUR)*, (2021), https://www.canada.ca/en/health-canada/services/drug-health-product-review-approval/submissions-under-review.html (accessed July 6, 2022).

58 Internet Archive: Wayback Machine, https://archive.org/web/ (accessed Sep. 1, 2021).

59 Health Canada Government of Canada, *The Drug and Health Product Register*, (2021), https://hpr-rps.hres.ca/index.php (accessed July 6, 2022).

60 WHO, *Anatomical Therapeutic Chemical (ATC) Classification*, https://www.who.int/tools/atc-ddd-toolkit/atc-classification (accessed July 6, 2022).

61 Health Canada Government of Canada, *Guidance Document: Notice of Compliance with Conditions (NOC/c)*, (2016), https://www.canada.ca/content/dam/hc-sc/migration/hc-sc/dhp-mps/alt_formats/pdf/prodpharma/applic-demande/guide-ld/compli-conform/noccg_accd-eng.pdf (accessed July 4, 2022).; Health Canada Government of Canada, *Guidance for Industry - Priority Review of Drug Submissions*, (2009), https://www.canada.ca/content/dam/hc-sc/migration/hc-sc/dhp-mps/alt_formats/hpfb-dgpsa/pdf/prodpharma/priordr-eng.pdf (accessed July 6, 2022).

62 Rita Banzi et al., *Approvals of Drugs With Uncertain Benefit–Risk Profiles in Europe*, 26 Eur J Intern Med 572–84 (2015).

63 HepaGam B (hepatitis B Immune Globulin) was conditionally authorized on Jan. 19, 2007 for prevention of hepatitis B recurrence following liver transplantation in adult patients with hepatitis B. There is no record of the conditions being removed.

64 Richard Pazdur, *Endpoints for Assessing Drug Activity in Clinical Trials*, 13 The Oncologist 19–21 (2008).
Food and Drug Regulations, C.08.002(h).

65
*Food and Drug Regulations*, CRC, c.870, s C.08.002(h); Health Canada Government of Canada, *Guidance for Industry - Priority Review of Drug Submissions*, (2009), https://www.canada.ca/content/dam/hc-sc/migration/hc-sc/dhp-mps/alt_formats/hpfb-dgpsa/pdf/prodpharma/priordr-eng.pdf (accessed July 6, 2022).

66 Joel Lexchin, *Notice of Compliance With Conditions: A Policy In Limbo*, 2 Healthc Policy 114–22 (2007); Joel Lexchin, *Quality of Evidence Considered by Health Canada in granting Full Market Authorisation to New Drugs with a Conditional Approval: A Retrospective Cohort study*, 8 BMJ Open e020377 (2018).; Jessica Pace et al., *Consumer Perspectives of Accelerated Access to Medicines: A Qualitative Study*, ahead-of-print J Health Organ Manag (2021).; Michael R. Law, *The Characteristics and Fulfillment of Conditional Prescription Drug Approvals in Canada*, 116 Health Policy 154–61 (2014).; H.-G. Eichler et al., *Adaptive Licensing: Taking the Next Step In the Evolution of Drug Approval*, 91 Clin Pharmacol Ther 426–37 (2012).

67 Health Canada Government of Canada, *Public Release of Clinical Information: guidance document*, (2019), https://www.canada.ca/en/health-canada/services/drug-health-product-review-approval/profile-public-release-clinical-information-guidance/document.html (accessed July 6, 2022).

68 James Leong Wai Yeen, Sam Salek & Stuart Walker, *Strategy for Communicating Benefit–Risk Decisions: a Comparison of Regulatory Agencies’ Publicly Available Documents*, 5 Front Pharmacol 269 (2014).; Andrea Keyter et al., *Can Standardisation of the Public Assessment Report Improve Benefit–Risk Communication?*, 11 Front. Pharmacol. 855 (2020).; Stuart Walker et al., *A Universal Framework for the Benefit–Risk Assessment of Medicines: Is This the Way Forward?*, 49 Ther Innov Regul Sci 17–25 (2015).; Neil McAuslane et al., *The Benefit–Risk Assessment of Medicines: Experience of a Consortium of Medium-Sized Regulatory Authorities*, 51 Ther Innov Regul Sci 635–44 (2017).

69 Joel Lexchin, *Health Canada’s Use of Accelerated Review Pathways And Therapeutic Innovation, 1995–2016: Cross-Sectional Analysis*, 8 BMJ Open e023605 (2018).; Joel Lexchin, *Health Canada’s Use of its Notice of Compliance With Conditions Drug Approval Policy: A Retrospective Cohort Analysis*, 49 Int J Health Serv 294–305 (2019).

70 Eric Lu et al., *What Constitutes an ‘Unmet Medical Need’ in Oncology? An Empirical Evaluation of Author Usage in the Biomedical Literature*, 44 Seminars in Oncology 8–12 (2017).

71 Melanie McPhail, Emma Weiss & Tania Bubela, *Conditional Drug Approval as a Path to Market for Oncology Drugs in Canada: Challenges and Recommendations for Assessing Eligibility and Regulatory Responsiveness*, 8 Front. Med. 818647 (2022).

72 Melanie McPhail, Emma Weiss & Tania Bubela, *Conditional Drug Approval as a Path to Market for Oncology Drugs in Canada: Challenges and Recommendations for Assessing Eligibility and Regulatory Responsiveness*, 8 Front. Med. 818647 (2022).

73 Health Canada Government of Canada, *Guidance for Industry - Priority Review of Drug Submissions*, (2009), https://www.canada.ca/content/dam/hc-sc/migration/hc-sc/dhp-mps/alt_formats/hpfb-dgpsa/pdf/prodpharma/priordr-eng.pdf (accessed July 6, 2022).

74 Health Canada Government of Canada, *Guidance Document: Notice of Compliance with Conditions (NOC/c)*, (2016), https://www.canada.ca/content/dam/hc-sc/migration/hc-sc/dhp-mps/alt_formats/pdf/prodpharma/applic-demande/guide-ld/compli-conform/noccg_accd-eng.pdf (accessed July 4, 2022).

75 Naci Huseyin, Katelyn R. Smalley & Aaron S. Kesselheim, *Characteristics of Preapproval and Postapproval Studies for Drugs Granted Accelerated Approval by the US Food and Drug Administration*, 318 JAMA 626–36 (2017).

76 Julia A. Beaver et al. *A 25-Year Experience of US Food and Drug Administration Accelerated Approval of Malignant Hematology and Oncology Drugs and Biologics: A Review*. JAMA Oncol 4, 849–856, doi: 10.1001/jamaoncol.2017.5618 (2018).

77 Aviv Ladanie et al., *Single Pivotal Trials With Few Corroborating Characteristics Were Used for FDA Approval of Cancer Therapies*, 114 J Clin Epidemiol 49–59 (2019).

78 Maximillian Salcher-Konrad, Huseyin Naci & Courtney Davis, *Approval of Cancer Drugs with Uncertain Therapeutic Value: A Comparison of Regulatory Decisions in Europe and the United States*, 98 The Millbank Quarterly 1219–1256 (2020).

79 Joel Lexchin, *Quality of evidence considered by Health Canada in Granting Full Market Authorisation to New Drugs With a Conditional Approval: A Retrospective Cohort Study*, 8 BMJ Open e020377 (2018).; Bishal Gyawali, Spencer Phillips Hey & Aaron S. Kesselheim, *Assessment of the Clinical Benefit of Cancer Drugs Receiving Accelerated Approval*, 179 JAMA Intern Med 906 (2019).

80 U. S. Government Accountability Office, *New Drug Approval: FDA Needs to Enhance Its Oversight of Drugs Approved on the Basis of Surrogate Endpoints*, (2009), https://www.gao.gov/products/gao-09-866 (accessed July 6, 2022).

81 21^st^Century Cures Act, Pub. L. No. 114–255, tit. IV, 130 Stat 1033 (2016) (codified at 42 USC § 201).

82 Bishal Gyawali, Spencer P. Hey & Aaron S. Kesselheim, *Evaluating the Evidence Behind the Surrogate Measures Included in the FDA’s Table of Surrogate Endpoints as Supporting Approval of Cancer Drugs*, 21 EClinicalMedicine 100332 (2020).

83 S. K. Andersen et al., *Conditional Approval of Cancer Drugs in Canada: Accountability And Impact on Public Funding*, 26 Curr Oncol e100–e105 (2019).

84 Bishal Gyawali, Spencer P. Hey & Aaron S. Kesselheim, *Evaluating the Evidence Behind the Surrogate Measures Included in the FDA’s Table of Surrogate Endpoints as Supporting Approval of Cancer Drugs*, 21 EClinicalMedicine 100332 (2020).; Vinay Prasad et al., *The Strength of Association Between Surrogate End Points and Survival in Oncology: A Systematic Review of Trial-Level Meta-analyses*, 175 JAMA Intern Med 1389 (2015).

85 Anna Kaltenboeck, Amanda Mehlman & Steven D. Pearson, *Strengthening the Accelerated Approval Pathway: An Analysis of Potential Policy Reforms and their Impact on Uncertainty, Access, Innovation, and Costs [White Paper.]*, (2021), https://icer.org/wp-content/uploads/2021/04/Strengthening-the-Accelerated-Approval-Pathway-_-ICER-White-Paper-_-April-2021.pdf (accessed July 7, 2022).; Bishal Gyawali, Spencer Phillips Hey & Aaron S. Kesselheim, *Assessment of the Clinical Benefit of Cancer Drugs Receiving Accelerated Approval*, 179 JAMA Intern Med 906 (2019).

86 Alison M Pease et al., *Postapproval Studies of Drugs Initially Approved by the FDA on the Basis of Limited Evidence: Systematic Review*, BMJ j1680 (2017).

87 Chul Kim & Vinay Prasad, *Cancer Drugs Approved on the Basis of a Surrogate End Point and Subsequent Overall Survival: An Analysis of 5 Years of US Food and Drug Administration Approvals*, 175 JAMA Intern Med 1992 (2015).

88 Bishal Gyawali, Spencer Phillips Hey & Aaron S. Kesselheim, *Assessment of the Clinical Benefit of Cancer Drugs Receiving Accelerated Approval*, 179 JAMA Intern Med 906 (2019).

89 Joel Lexchin, *Quality of Evidence Considered by Health Canada in Granting Full Market Authorisation to New Drugs With a Conditional Approval: A Retrospective Cohort Study*, 8 BMJ Open e020377 (2018).

90 Bishal Gyawali, Spencer P. Hey & Aaron S. Kesselheim, *Evaluating the Evidence Behind the Surrogate Measures Included in the FDA’s Table of Surrogate Endpoints as Supporting Approval of Cancer Drugs*, 21 EClinicalMedicine 100332 (2020).

91 Anna Kaltenboeck, Amanda Mehlman & Steven D. Pearson, *Strengthening the Accelerated Approval Pathway: An Analysis of Potential Policy Reforms and their Impact on Uncertainty, Access, Innovation, and Costs [White Paper.]*, (2021), https://icer.org/wp-content/uploads/2021/04/Strengthening-the-Accelerated-Approval-Pathway-_-ICER-White-Paper-_-April-2021.pdf (accessed July 7, 2022).; Committee on Ethical and Scientific Issues in Studying the Safety of Approved Drugs, Board on Population Health and Public Health Practice, & Institute of Medicine, Ethical and Scientific Issues in Studying the Safety of Approved Drugs (2012), http://www.ncbi.nlm.nih.gov/books/NBK200905/ (accessed July 6, 2022).

92 Canadian Agency for Drugs and Technologies in Health, *Real-World Evidence: A Primer* (2023) https://www.cadth.ca/real-world-evidence-primer (accessed Jan. 30, 2023) [distinguishing between real-world data (information collected outside of traditional clinical trials) and real-world evidence (evidence derived from real-world data)]; United States Food and Drug Administration, *Real-World Evidence* (2023) https://www.fda.gov/science-research/science-and-research-special-topics/real-world-evidence (accessed Jan. 30, 2023) [defining real-world data as data relating to patient health status and the delivery of health care and real-world evidence as clinical evidence derived from analysis of real-world data].

93
*Protecting Canadians from Unsafe Drugs Act (Vanessa’s Law)*, SC 2014, c.24.

94
*Food and Drugs Act*, RSC, 1985, c. F-27, ss 21.31–3.2.

95
Government of Canada, Health Canada, Amendments to the Food and Drugs Act: guide to new authorities (power to require and disclose information, power to order a label change, power to order a recall, power to require assessments, and power to require tests, studies, etc.) (2021), https://epe.lac-bac.gc.ca/100/201/301/weekly_acquisitions_list-ef/2021/21-12/publications.gc.ca/collections/collection_2021/sc-hc/H164-307-2021-eng.pdf (accessed July 6, 2022).

96 Anna Kaltenboeck, Amanda Mehlman & Steven D. Pearson, *Strengthening the Accelerated Approval Pathway: An Analysis of Potential Policy Reforms and their Impact on Uncertainty, Access, Innovation, and Costs [White Paper]*, (2021), https://icer.org/wp-content/uploads/2021/04/Strengthening-the-Accelerated-Approval-Pathway-_-ICER-White-Paper-_-April-2021.pdf (accessed July 7, 2022).

97
Committee on Ethical and Scientific Issues in Studying the Safety of Approved Drugs, Board on Population Health and Public Health Practice, & Institute of Medicine, Ethical and Scientific Issues in Studying the Safety of Approved Drugs (2012), http://www.ncbi.nlm.nih.gov/books/NBK200905/ (accessed July 6, 2022).

98 U. S. Government Accountability Office, *New Drug Approval: FDA Needs to Enhance Its Oversight of Drugs Approved on the Basis of Surrogate Endpoints*, (2009), https://www.gao.gov/products/gao-09-866 (accessed July 6, 2022).

99 Health Canada Government of Canada, *Notice of Compliance with conditions (NOC/c)*, (2021), https://www.canada.ca/en/health-canada/services/drugs-health-products/drug-products/notice-compliance/conditions.html (accessed July 6, 2022); *Food and Drug Regulations*, CRC 870, s C.08.006(2)(a).

100 Health Canada Government of Canada, *Archived – Safety Information Regarding Selective COX-2 Inhibitor NSAIDs: Vioxx, (rofecoxib), Celebrex (celecoxib), Bextra (valdecoxib), Mobicox (meloxicam) and Generic Forms of Meloxicam - Recalls, Advisories and Safety Alerts – Canada.ca*, (2013), https://recalls-rappels.canada.ca/en/alert-recall/archived-safety-information-regarding-selective-cox-2-inhibitor-nsaids-vioxx-rofecoxib (accessed July 6, 2022).

101 Government of Canada, Health Canada, *Clarification from Health Canada Regarding the Status of Iressa (gefitinib) in Canada*, (2005), http://web.archive.org/web/20080630163440/http://www.hc-sc.gc.ca/dhp-mps/prodpharma/activit/fs-fi/fact_iressa-eng.php (accessed July 6, 2022).

102 Health Canada Government of Canada, *Regulatory Decision Summary - ICLUSIG - Health Canada*, (2021), https://hpr-rps.hres.ca/reg-content/regulatory-decision-summary-detail.php?linkID=RDS00083 (accessed July 6, 2022).

103 Melanie McPhail, Emma Weiss & Tania Bubela, *Conditional Drug Approval as a Path to Market for Oncology Drugs in Canada: Challenges and Recommendations for Assessing Eligibility and Regulatory Responsiveness*, 8 Front. Med. 818647 (2022).

104 Michael R. Law, *The Characteristics and Fulfillment of Conditional Prescription Drug Approvals in Canada*, 116 Health Policy 154–61 (2014).; Bishal Gyawali & Aaron S. Kesselheim, *Reinforcing the Social Compromise of Accelerated Approval*, 15 Nat Rev Clin Oncol 596–97 (2018).

105 NOC/c and Priority Review Working Group, *Notice of Compliance with Conditions Policy Evaluation - Draft*, (2000), https://s3.amazonaws.com/s3.documentcloud.org/documents/269455/nocc-review-ati-docs.pdf (accessed July 6, 2022).

106 Anna Kaltenboeck, Amanda Mehlman & Steven D. Pearson, *Strengthening the Accelerated Approval Pathway: An Analysis of Potential Policy Reforms and their Impact on Uncertainty, Access, Innovation, and Costs [White Paper.]*, (2021), https://icer.org/wp-content/uploads/2021/04/Strengthening-the-Accelerated-Approval-Pathway-_-ICER-White-Paper-_-April-2021.pdf (accessed July 7, 2022).; Bishal Gyawali & Aaron S. Kesselheim, *Reinforcing the Social Compromise of Accelerated Approval*, 15 Nat Rev Clin Oncol 596–97 (2018).; Julia A. Beaver & Richard Pazdur, *‘Dangling’ Accelerated Approvals in Oncology*, 384 N Engl J Med e68 (2021). Jonathan J. Darrow, *Few New Drugs Deserve Expedited Regulatory Treatment*, 27 JMCP 685–88 (2021).; Elisabeth Mahase, *FDA Allows Drugs Without Proven Clinical Benefit to Languish for Years on Accelerated Pathway*, 374 BMJ n1898 (2021).

107 Health Canada, *LARTRUVO (olaratumab) – New Clinical trial information important to prescribing decisions* (2019) https://recalls-rappels.canada.ca/en/alert-recall/lartruvo-olaratumab-new-clinical-trialinformation- important-prescribing-decisions (accessed Jan. 30, 2023).

108 Bishal Gyawali & Aaron S. Kesselheim, *Reinforcing the Social Compromise of Accelerated Approval*, 15 Nat Rev Clin Oncol 596–97 (2018).

