# Supplementary material for: Lessons from Canada’s notice of compliance with conditions policy for the life-cycle regulation of drugs
Source: J Law Biosci. 2023 Apr 12;10(1):lsad008. doi: 10.1093/jlb/lsad008 (PMC10101551; doi:10.1093/jlb/lsad008)
Supplement: Appendix_1_lsad008 [file appendix_1_lsad008.docx]

Appendix 1: Total list of drug-indications authorized under the NOC/c policy 1998-2021

| **Medicinal ingredient** | **Date NOC/c granted** | **Date Conditions Met** | **Authorized Indication** |
| --- | --- | --- | --- |
| 1. delavirdine | 7/22/1998 | 7/22/2003 | not specified |
| 1. nevirapine | 9/4/1998 | 9/13/2004 | not specified |
| 1. recombinant factor VII activated | 2/12/1999 | 5/19/2006 | bleeding episodes in patients who have hemophilia A or B with inhibitors to clotting factors VIII or IX |
| 1. alteplase | 2/16/1999 | 1/26/2005 | management of acute ischemic stroke in adults to improve neurological recovery and reduce disability |
| 1. abacavir sulfate | 6/4/1999 | 9/10/2001 | not specified |
| 1. zanamivir | 11/2/1999 | 8/26/2003 | Treatment of influenza in paediatrics 7 years and older |
| 1. riluzole | 8/30/2000 | 11/29/2007 | Amyotrophic lateral sclerosis |
| 1. amprenavir | 3/1/2001 | 7/5/2004 | not specified |
| 1. imatinib | 9/20/2001 | 12/29/2004 | Adult patients with Philadelphia chromosome-positive CML in blast crisis, accelerated phase or in chronic phase (after failure of interferon-ax therapy). |
| 1. tenofovir disoproxil fumarate | 3/18/2003 | 7/20/2005 | treatment of HIV-1 infection in combination with other antiretroviral agents in patients 18 years and older who have experienced virologic failure on other regimens |
| 1. bicalutamide | 8/13/2003 | 8/13/2003 | for immediate therapy in some patients with localized (T1-T2) prostate cancer who are inappropriate for surgery or radiotherapy |
| 1. imatinib | 10/8/2003 | 6/17/2010 | Adult patients with newly diagnosed Philadelphia chromosome positive Chronic Myeloid Leukemia |
| 1. gefitinib | 12/17/2003 | 12/18/2009 | third line treatment of patients with locally advanced or metastatic NSCLC after failure of two prior chemotherapy regimens |
| 1. agalsidase alfa | 2/6/2004 | 6/20/2004 | enzyme replacement therapy for fabry disease |
| 1. celecoxib | 5/20/2004 | 12/17/2004 | treatment/prevention of familial adenomatous polyposis |
| 1. anastrozole | 6/30/2004 | 12/2/2008 | adjuvant treatment of postmenopausal women with hormone receptor positive early breast cancer |
| 1. memantine hydrochloride | 12/8/2004 | 4/21/2011 | treatment of patients with moderate to severe dementia of the Alzheimer’s type |
| 1. bortezomib | 1/27/2005 | 9/11/2007 | treatment of multiple myeloma patients who have relapsed following front-line therapy and refractory to their most recent therapy. Treatment must be initiated and administered under the supervision of a qualified healthcare professional who is experienced in the use of antineoplastic therapy |
| 1. letrozole | 4/1/2005 | 12/17/2010 | for the extended adjuvant treatment of early breast cancer in post-menopausal women who have received prior standard adjuvant tamoxifen therapy |
| 1. delta-9-tetrahydrocannabinol/cannabidiol | 4/15/2005 | 12/12/2019 | symptomatic relief of neuropathic pain in multiple sclerosis |
| 1. capecitabine | 12/7/2005 | 10/23/2008 | adjuvant treatment of patients with Stage III (Dukes' stage C) colon cancer |
| 1. exemestane | 5/12/2006 | 6/6/2008 | adjuvant treatment of early breast cancer |
| 1. sorafenib tablets | 7/28/2006 | 6/12/2009 | treatment of locally advanced/metastatic renal cell carcinoma in patients who failed prior cytokine therapy or are considered unsuitable for such therapy |
| 1. sunitinab malate | 8/17/2006 | 4/23/2010 | treatment of metastatic renal cell carcinoma of clear cell histology after failure of cytokine based therapy or in patients who are considered likely to be intolerant of such therapy |
| 1. letrozole | 10/6/2006 | 12/17/2010 | for the adjuvant treatment of post-menopausal women with hormone receptor positive early breast cancer |
| 1. deferasirox | 10/18/2006 | 12/2/2016 | management of chronic iron overload in patients with transfusion dependent anemias in patients 6 years or older or aged 2-5 who cannot be adequately treated with deferoxamine |
| 1. docetaxel | 12/14/2006 | 9/26/2012 | adjuvant treatment of patients with operable node-positive breast cancer, in combination with doxorubicin and cyclophosphamide |
| 1. hepatitis B Immune Globulin | 1/19/2007 | n/a | prevention of hepatitis B recurrence following liver transplantation in adult patients with hepatitis B who have no or low levels of HBV replication |
| 1. levodopa/carbidopa | 3/1/2007 | 3/12/2014 | advanced levodopa-responsive Parkinson's disease who do not have satisfactory control of motor fluctuations and hyper/dyskinesia despite optimized treatment with available combinations of PD medicinal products |
| 1. dasatinib | 3/26/2007 | 11/19/2009 | treatment of adults with chronic, accelerated or blast phase chronic myeloid leukemia with resistance or intolerance to prior therapy including imatinib mesylate |
| 1. imatinib mesylate | 5/24/2007 | 2/21/2013 | Treatment of pediatric patients with newly diagnosed Philadelphia chromosome-positive chronic myeloid leukemia in chronic phase |
| 1. delta-9-tetrahydrocannabinol/cannabidiol | 8/1/2007 | 12/12/2019 | adjuvant analgesic treatment in adult patients with advanced cancer who experience moderate to severe pain during the highest tolerated dose of strong opioid therapy for persistent background pain |
| 1. nelarabine | 9/22/2007 | 1/22/2020 | Patients with refractory or recurrent T-ALL or T-lymphoblastic lymphoma |
| 1. nesiritide | 11/8/2007 | 8/1/2012 | treatment of hospitalized patients with acute episodes of heart failure (HF) who did not respond to treatment with diuretic drugs for two hours, |
| 1. pregabalin | 11/9/2007 | 6/29/2010 | symptomatic relief of neuropathic pain associated with damaged nerves of the CNS including the brain and spinal cord |
| 1. raltegravir potassium | 11/27/2007 | 3/4/2009 | in combination with other antiretroviral agents, is indicated for the treatment of HIV-1 infection in treatment experienced adult patients who have evidence of viral replication and HIV-1 strains resistant to multiple antiretroviral agents. |
| 1. lenalidomide | 1/17/2008 | 6/6/2013 | treatment of patients with transfusion dependent anemia due to low or intermediate 1 risk myelodysplastic syndromes associated with deletion 5q cytogenic abnormality with or without additional cytogenetic abnormalities |
| 1. panitumumab | 4/3/2008 | 2/19/2015 | monotherapy for the treatment of patients with EGFR expressing metastatic colorectal carcinoma with non-mutated (wild type) KRAS after failure of fluoropyrimidine-, oxaliplatin- and irinotecan-containing chemotherapy regimens |
| 1. sunitinab malate | 5/1/2008 | 4/23/2010 | treatment of metastatic renal cell carcinoma of clear cell histology |
| 1. darunavir/TMC114 | 6/18/2008 | 2/11/2009 | co-administered with ritonavir and with other ARB agents for the treatment of HIV infection in treatment-experienced adult patients who have failed prior antiretroviral therapy |
| 1. idebenone | 7/23/2008 | 4/30/2013 | Friedrich's Ataxia |
| 1. nilotinib hydrochloride monohydrate | 9/9/2008 | 11/30/2011 | accelerated phase philadelphia chromosome-positive CML in adult patients resistant to or intolerant of at least one prior therapy including imatinib |
| 1. bevacizumab | 2/6/2009 | 11/25/2011 | used in combination with paclitaxel for treatment of patients with HER2 negative breast cancer and who are ECOG class 0-1 |
| 1. aztreonam for inhalation solution | 9/11/2009 | 5/17/2011 | for the management of cystic fibrosis patients with chronic pulmonary P.A. infections |
| 1. memantine hydrochloride | 10/29/2009 | 4/21/2011 | not specified |
| 1. memantine hydrochloride | 11/4/2009 | 4/21/2011 | not specified |
| 1. memantine hydrochloride | 11/25/2009 | 4/21/2011 | not specified |
| 1. imatinib mesylate | 12/22/2009 | 11/24/2017 | For the adjuvant treatment of adult patients who are at intermediate to high risk of relapse following complete resection of Kit (CD117) positive GIST |
| 1. bevacizumab | 3/24/2010 | 5/23/2018 | for the treatment of patients with GBM after relapse or disease progression |
| 1. letrozole | 4/7/2010 | 2/14/2013 | (1) adjuvant treatment of postmenopausal women with hormone receptor positive early breast cancer, and  (2) the extended adjuvant treatment of hormone receptor positive early breast cancer in postmenopausal women who have received approximately 5 yeras of prior stnadard adjuvant tamoxifen therapy |
| 1. memantine hydrochloride | 4/16/2010 | 12/20/2011 | symptomatic treatment of patients with moderate to severe dementia of the alzheimer's type |
| 1. letrozole | 4/28/2010 | 12/2/2011 | (1) adjuvant treatment of postmenopausal women with hormone receptor positive early breast cancer, and  (2) the extended adjuvant treatment of hormone receptor positive early breast cancer in postmenopausal women who have received approximately 5 yeras of prior stnadard adjuvant tamoxifen therapy |
| 1. letrozole | 4/28/2010 | 8/16/2011 | (1) adjuvant treatment of postmenopausal women with hormone receptor positive early breast cancer, and  (2) the extended adjuvant treatment of hormone receptor positive early breast cancer in postmenopausal women who have received approximately 5 yeras of prior stnadard adjuvant tamoxifen therapy |
| 1. letrozole | 4/28/2010 | 12/10/2013 | not specified |
| 1. letrozole | 4/28/2010 | 6/14/2011 | (1) adjuvant treatment of postmenopausal women with hormone receptor positive early breast cancer, and  (2) the extended adjuvant treatment of hormone receptor positive early breast cancer in postmenopausal women who have received approximately 5 yeras of prior stnadard adjuvant tamoxifen therapy |
| 1. letrozole | 4/28/2010 | 6/24/2011 | (1) adjuvant treatment of postmenopausal women with hormone receptor positive early breast cancer, and  (2) the extended adjuvant treatment of hormone receptor positive early breast cancer in postmenopausal women who have received approximately 5 yeras of prior stnadard adjuvant tamoxifen therapy |
| 1. letrozole | 4/28/2010 | 6/22/2011 | (1) adjuvant treatment of postmenopausal women with hormone receptor positive early breast cancer, and  (2) the extended adjuvant treatment of hormone receptor positive early breast cancer in postmenopausal women who have received approximately 5 yeras of prior stnadard adjuvant tamoxifen therapy |
| 1. memantine hydrochloride | 5/13/2010 | 12/21/2013 | symptomatic treatment of patients with moderate to severe dementia of the alzheimer's type |
| 1. nilotinib hydrochloride monohydrate | 7/22/2010 | 8/18/2011 | treatment of chronic phase Ph+ CML in adult patients resistant to or intolerant of at least one prior therapy including imatinib |
| 1. letrozole | 8/27/2010 | 5/20/2011 | (1) adjuvant treatment of postmenopausal women with hormone receptor positive early breast cancer, and  (2) the extended adjuvant treatment of hormone receptor positive early breast cancer in postmenopausal women who have received approximately 5 yeras of prior stnadard adjuvant tamoxifen therapy |
| 1. memantine hydrochloride | 10/5/2010 | 12/21/2011 | symptomatic treatment of patients with moderate to severe dementia of the Alzheimer's type |
| 1. letrozole | 12/9/2010 | 9/6/2013 | (1) adjuvant treatment of postmenopausal women with hormone receptor positive early breast cancer, and  (2) the extended adjuvant treatment of hormone receptor positive early breast cancer in postmenopausal women who have received approximately 5 yeras of prior stnadard adjuvant tamoxifen therapy |
| 1. docetaxel | 2/25/2011 | 10/10/2013 | for use in combination with doxorubicin and cyclophosphamide for the adjuvant treatment of patients with operable node positive breast cancer |
| 1. nilotinib hydrochloride monohydrate | 6/23/2011 | 8/19/2015 | treatment of adult patients with newly diagnosed Ph+ CML in chronic phase |
| 1. everolimus | 6/30/2011 | 4/29/2016 | for the treatment of patients of 3 years of age or older with subependymal giant cell astrocytoma (SEGA) associated with tuberous sclerosis complex that have demonstrated serial growth who are not candidates for surgical resection and for whom immediate surgical intervention is not required |
| 1. dasatinib | 7/19/2011 | 11/26/2015 | treatment of adults with newly diagnosed Ph+ CML in chronic phase |
| 1. ofatumumab | 3/9/2012 | n/a | chronic lymphocytic leukemia refractory to fludarabine and alemtuzumab |
| 1. crizotinib | 4/25/2012 | 11/18/2015 | monotherapy in patients with ALK postiive locally advanced or metastatic NSCLC |
| 1. remestemcel-L | 5/17/2012 | n/a | acute graft versus host disease in pediatric patients ; refractory to treatment with systemic corticosteroid therpay and/or other immunosuppressive agents |
| 1. everolimus | 1/25/2013 | 9/23/2016 | the treatment of adult patients with renal angiomyolipoma associated with tuberous sclerosis complex who do not require immediate surgery |
| 1. brentuximab vedotin | 2/1/2013 | 5/12/2020 | (1) patients with Hodgkin lymphoma (HL) after failure of autologous stem cell transplant (ASCT) or after failure of at least two multi-agent chemotherapy regimens in patients who are not ASCT candidates;  (2) patients with systemic anaplastic large cell lymphoma after failure of at least one multi-agent chemotherapy regimen |
| 1. eculizumab | 3/1/2013 | 6/30/2015 | treatment of patients with atypical hemolytic uremic syndrome to reduce complement-mediated thrombotic microangiopathy in children less than 13 yeras of age and/or weighing less than 40 kg [no conditions for adults] |
| 1. romidepsin | 10/16/2013 | n/a | patients with relapsed/refractory peripheral T-cell lymphoma who are not eligible for transplant and have received at least one prior systemic therapy |
| 1. bosutinib | 3/7/2014 | 8/2/2017 | the treatment of chronic, accelerated, or blast phase Ph+ Chronic Myelogenous Leukemia in adult patients with resistance or intolerance to prior tyrosine kinase inhibitor therapy, and for whom subsequent treatment with imatinib, nilotinib, and dasatinib is not clinically appropriate |
| 1. imatinib mesylate | 4/24/2014 | 5/26/2014 | not specified |
| 1. imatinib mesylate | 7/7/2014 | 7/7/2014 | not specified |
| 1. simeprevir | 1/30/2015 | 11/29/2016 | in combination with sofosbuvir (and without the need for peginterferon alfa and ribavirin) for the treatment of genotype 1 chronic hepatitis C in adults with compensated liver disease |
| 1. dabrafenib | 3/6/2015 | 5/13/2016 | in combination with trametinib for the treatment of patients with unresectable or metastatic melanoma with a BRAF V600 mutation |
| 1. idelalisib | 3/27/2015 | 4/21/2020 | monotherapy for the treatment of patients with follicular lymphoma who have received at least two prior systemic regimens and are refractory to both rituximab and an alkylating agent |
| 1. ceritinib | 3/27/2015 | n/a | anaplastic lyphoma kinase positive locally advanced or metastatic non-small cell lung cancer who have progressed on or who were intolerant to crizotinib |
| 1. ponatinib hydrochloride | 4/2/2015 | n/a | adult patients with chronic phase, accelerated phase, or blast phase chronic myeloid leukemia or Ph+ acute lymphoblastic leukemia for whom other tyrosine kinase inhibitor therapy is not appropriate including if T3151 mutation position or prior TKI resistance or intolerance |
| 1. pembrolizumab | 5/19/2015 | 12/22/2017 | unresectable or metastatic melanoma and disease progression following ipilimumab therapy, and if BRAF V600 mutation positive, following a BRAF or MEK inhibitor |
| 1. ibrutinib | 7/28/2015 | 9/12/2017 | relapsed or refractory mantle cell lymphoma |
| 1. daclatasvir | 8/13/2015 | 11/9/2016 | for use in combination with other agents for the treatment of chronic hepatitis in adult patients with hepatitis C virus genotype 3 and compensated liver disease including cirrhosis |
| 1. asfotase alfa | 8/14/2015 | n/a | enzyme replacement therapy for patients with confirmed diagnosis of paediatric-onset hypophosphatasia |
| 1. blinatumomab | 12/22/2015 | 11/16/2017 | treatment of adults with Philadelphia chromosome-negative relapsed or refractory B precursor acute lymphoblastic leukemia (ALL). |
| 1. deferasirox | 2/24/2016 | 1/10/2017 | (1) the management of chronic iron overload in patients with transfusion dependent anemias aged six years or older (2) the management of chronic iron overload in patients with transfusion dependent anemias aged two to dfive who cannot be adequately treated with deferoxamine |
| 1. trametinib | 3/11/2016 | 5/13/2016 | in combination with dabrafenib for the treatmnet of patients with unresectable or metastatic melanoma with a BRAF V600 mutation |
| 1. palbociclib | 3/16/2016 | 11/17/2017 | For use in combination with letrozole for the treatment of postmenopausal women with estrogen-receptor positive human epidermal growth factor receptor 2 negative advanced breast cancer as initial endocrine based therapy for their metastatic disease |
| 1. pembrolizumab | 4/15/2016 | 9/10/2019 | treatment of patients with metastatic NSCLC whose tumours express PD-L-1 and who have disease progression on or after platinum containing chemotherapy. Patients with EGFR or ALK genomic tumour abberations should have disease progression on authorized therapy for these abberations prior to receiving Keytruda. |
| 1. olaparib | 4/29/2016 | 5/2/2018 | Maintenance treatment of adult patients with platinum-sensitive relapsed BRCA mutated high grade serous epithelial ovarian, fallopian tube or primary peritoneal cancer who are in response to platinum based chemotherapy |
| 1. nivolumab | 4/29/2016 | 2/21/2018 | treatment of patients with unresectable or metastatic melanoma and disease progression following ipilimumab and, if BRAF V600 positive, a BRAF Inhibitor |
| 1. idarucizumab | 4/29/2016 | 6/28/2018 | antidote specific for dabigatran in patients treated with Pradaza when rapid specific reversal of the anticoagulant effects of dabigatran is required either for emergency surgery/urgent procedures or in situations of life--threatening or uncontrolled bleeding |
| 1. daratumumab | 6/29/2016 | 10/10/2018 | patients with multiple myeloma who have received at least three prior lines of therapy including a proteasome inhibitor and an immunodulatory agent, or who are refractory to both a PI and an IMiD |
| 1. osimertinib | 7/5/2016 | 1/19/2018 | patients with locally advanced or metastatic EGFR T790M mutation-positive NSCLC who have progressed on or after EGFR tKI therapy. Validaed test is required to identify EGFR T790M mutation-positive status prior to treatment |
| 1. alectinib | 9/29/2016 | 9/26/2018 | monotherapy for the treatment of patients with ALK-positive locally advanced or metastatic NSCLC who have progressed or are intolerant to crizotinib |
| 1. venetoclax | 9/30/2016 | 1/13/2020 | monotherapy for the treatment of patients with Chronic lymphocytic leukemia (CLL) with 17p deletion who have received at least one prior therapy or patients with CLL without 17p deletion who have received at least one prior therapy and for whom there are no other available treatment options |
| 1. nivolumab | 10/26/2016 | n/a | (1) treatment of patients with unresectable or metastatic BRAF V600 mutation position melanoma in prviously untreated adults;  (2) treatment of patients with unresectable or metastatic melanoma in previously untreated adults when used in combination with ipilimumab [relative to opdivo monotherapy an increase in PFS for the combination of opdivo and ipilimumab is established only in patietns with low tumour PD-L1 expression |
| 1. agalsidase alfa | 2/10/2017 | n/a | long-term enzyme replacement therapy in patients with confirmed diagnosis of Fabry disease |
| 1. thiotepa | 3/29/2017 | n/a | As part of a high dose chemotherapy consolidation regimen followed by ASTC in adult patients with CNS lymphoma |
| 1. atezolizumab | 4/12/2017 | n/a | patients with locally advanced or metastatic urothelial carcinoma who: Have disease progression during or following platinum-containing chemotherapy Have disease progression within 12 months of neoadjuvant or adjuvant treatment with platinum containing chemotherapy, |
| 1. blinatumomab | 4/28/2017 | n/a | Indicated for the treatment of pediatric patients with Philadelphia chromosome-negative relapsed or refractory B cell precursor ALL |
| 1. obeticholic acid | 5/24/2017 | n/a | primary biliary cholangitis in combination with ursodeoxycholic acid in adults with an inadequate response to UDCA or as monotherapy in adults unable to tolerate UDCA |
| 1. pembrolizumab | 9/8/2017 | n/a | refractory or relapsed classical hodgkin lymphoma (CHL) as monotherapy in adults who have failed autologous stem cell transplant and brentouximab vedotin or who are not asct candidates and have have failed brentouximab vedotin |
| 1. durvalumab for injection | 11/3/2017 | n/a | patients with locally advanced or metastatic urothelial carcinoma who have disease progression during or following platinum containing chemotherapy or have disease progression within 12 months of neoadjuvant or adjuvant threatment with platinum-containing chemotherapy |
| 1. nivolumab | 11/10/2017 | n/a | adult patients with classical Hodgkin Lymphoma that has relapsed or progressed after autologous stem cell transplantation and brentuximab vedotin, or 3 or more lines of systemic therapy including ASCT |
| 1. olaratumab | 11/23/2017 | n/a | In combination with doxorubicin for the treatment of adult patients with advanced soft tissue sarcoma not amenable to curative treatment with radiotherapy or surgery and for whom treatment with an anthracycline-containing regimen is appropriate |
| 1. sebelipase alfa | 12/15/2017 | n/a | treatment of lysosomal acid lipase deficiency (LALD) |
| 1. avelumab | 12/17/2017 | n/a | treatment of patients with metastatic Merkel cell carcinoma in previously treated adults |
| 1. ocrelizumab | 2/14/2018 | n/a | management of adult patients with early primary progressive multiple sclerosis (PPMS) as defined by disease duration and level of disability, in conjunction with imaging features characteristic of inflammatory activity |
| 1. nivolumab | 3/23/2018 | n/a | monotherapy for the treatment of adult patients with advanced (not amenable to curative therapy or local therapeutic measures) or metastatic hepatocellular carcinoma (HCC) who are intolerant to or have progressed on sorafenib therapy |
| 1. avelumab | 5/3/2018 | 5/14/2019 | locally advanced or metastatic urothelial carcinoma who have received prior platinum-based chemotherapy |
| 1. durvalumad | 5/4/2018 | 8/23/2019 | locally advanced unresectable NSCLC whose disease has not progressed following platinum based chemoradiation therapy |
| 1. olaparib | 5/4/2018 | n/a | monotherapy for the maintenance treatment of adult patients with platinum-sensitive relapsed (PSR) BRCA wild type high-grade epithelial ovarian, fallopian tube, or primary peritoneal cancer who are in response (complete response or partial response) to platinum-based chemotherapy |
| 1. brigatinib | 7/26/2018 | n/a | adult patients with anaplastic lymphoma kinase positive metastatic non-small cell lung cancer who have progressed on or were intolerant to an ALK inhibitor (crizotinib) |
| 1. pembrolizumab | 9/21/2018 | n/a | adult and pediatric patients with relapsed or refractory Primary Mediastinal B-cell Lymphoma (rrPMBCL) or who have relapsed after 2 or more lines of therapy, as monotherapy |
| 1. pralatrexate | 10/26/2018 | n/a | patients with relapsed or refractory peripheral T-cell lymphoma (PTCL) |
| 1. enasidenib (as enasidenib mesylate) | 2/6/2019 | n/a | treatment of adult patients with relapsed or refractory AML with an isocitrate dehydrogenase-2 IHG2 mutation |
| 1. lorlatinib | 2/22/2019 | n/a | monotherapy for the treatment of adult patients with ALK-positive metastatic NSCLC who have progressed on crizotinib and at least one other ALK inhibitor, or patients who have progressed on ceritinib or alectinib |
| 1. cemiplimab | 4/10/2019 | n/a | treatment of adult patients with metastatic or locally advanced cutaneous squamous cell carcinoma who are not candidates for curative surgery or curative radiation |
| 1. pembrolizumab | 4/11/2019 | n/a | treatment of patients with locally advanced unresectable or metastatic urothelial carcinoma, as monotherapy, in adults who are not eligible for cisplatin-containing chemotherapy and whose tumors express PD-L1 [Combined Positive Score (CPS) ≥10] as determined by a validated test, or in adults who are not eligible for any platinum-containing chemotherapy regardless of PD-L1 status, |
| 1. pembrolizumab | 4/18/2019 | n/a | adults with (1) unresectable or metastic MSI-H or dMMR colorectal cancer that has progressed following treatment with a fluoropyrimidine, oxaliplatin, and irinotecan, or (2) MSI-H or dMMR endometrial cancer that has progressed following prior therapy and who have no satisfactory alternative treatment options |
| 1. larotrectinib | 7/10/2019 | n/a | treatment of adult and pediatric patients with solid tumours that have NTRK gene fusion without a known acquired resistance mutation; are metastatic or where surgical resection is likely to result in severe morbidity; and have no satisfactory treatment options |
| 1. atezolizumab | 8/21/2019 | n/a | in combination with nab-paclitaxel, for the treatment of adult patients with unresectable, locally advanced or metastatic triple-negative breast cancer (TNBC) whose tumours have PD-L1 expression ≥ 1%, and who have not received prior chemotherapy for metastatic disease |
| 1. pembrolizumab | 9/20/2019 | n/a | treatment of adult patients, in combination with lenvatinib, with advanced endometrial carcinoma that is not MSI-H or dMMR who have disease progression following prior platinum-based therapy and are not candidates for curative surgery or radiation |
| 1. lenvatinib mesylate | 9/20/2019 | n/a | in combination with pembrolizumab, for the treatment of adult patients with advanced endometrial carcinoma that is not MSI-H or dMMR, who have disease progression following prior platinum-based systemic therapy, and are not candidates for curative surgery or radiation |
| 1. erdafitinib | 10/25/2019 | n/a | adult patients with locally advanced or metastatic urothelial carcinoma (UC) whose tumors have susceptible FGFR2 or FGFR3 genetic alterations and; who have disease progression during or following at least one line of prior chemotherapy, including within 12 months of neoadjuvant or adjuvant chemotherapy, |
| 1. avelumab | 11/5/2019 | n/a | treatment of adult patients with metastatic Merkel cell carcinoma |
| 1. blinatumomab | 12/19/2019 | n/a | Indicated for the treatment of patients with Philadelphia chromosome-negative CD19 positive B-precursor ALL, in first or second hematologic complete remission with minimal residue disease greater or equal to 0.1%/ treatment of patients with minimal residual disease positive B-cell precursor ALL |
| 1. entrectinib | 2/10/2020 | n/a | the treatment of adult patients with unresectable locally advanced or metastatic extracranial solid tumours, including brain metastases, that have a neurotrophic tyrosine receptor kinase (NTRK) gene fusion without a known acquired resistance mutation, and with no satisfactory treatment options |
| 1. polatuzumab vedotin | 7/9/2020 | n/a | in combination with bendamustine and rituximab for the treatment of adult patients with relapsed or refractory diffuse large B-cell lymphoma, not otherwise specified, who are not eligible for ASCT and have received at least one prior therapy |
| 1. remdesivir | 7/27/2020 | n/a | for the treatment of coronavirus disease 2019 (COVID-19) in adults and adolescents (aged 12 years and older with body weight at least 40 kg) with pneumonia requiring supplemental oxygen |
| 1. pembrolizumab | 12/14/2020 | n/a | KEYTRUDA IS INDICATED FOR THE TREATMENT OF ADULT PATIENTS WITH BACILLUS CALMETTE-GUERIN (BCG)-UNRESPONSIVE, HIGH-RISK, NON-MUSCLE INVASIVE BLADDER CANCER (NMIBC) WITH CARCINOMA IN-SITU (CIS) WITH OR WITHOUT PAPILARY TUMOURS WHO ARE ELIGIBLE FOR OR HAVE ELECTED NOT TO UNDERGO CYSTECTOMY |
| 1. Bacillus Calmette-Guérin (BCG): Russian BCG-I (Russian or Moscow) strain | 12/24/2020 | n/a | adjuvant therapy after transurethral resection (TUR) of a primary or relapsing superficial papillary urothelial cell carcinoma of the bladder stage Ta (grade 2 or 3) or T1 (grade 1, 2, or 3), without concomitant carcinoma in situ. It is only recommended for stage Ta grade 1 papillary tumors, when there is judged to be a high risk (>50%) of tumor recurrence. |
| 1. pembrolizumab | 2/5/2021 | n/a | the treatment of adult and pediatric patients with refractory or relapsed classical Hodgkin Lymphoma (cHL) who have failed autologous stem cell transplant (ASCT), or who are not candidates for multi-agent salvage chemotherapy and ASCT, |
| 1. nivolumab | 2/11/2021 | n/a | IN COMBINATION WITH IPILIMUMAB IS INDICATED FOR THE TREATMENT OF ADULT PATIENTS WITH MICROSATELLITE INSTABILOITY-HIGH (MSI-H) OR MISMATCH REPAIR DEFICIENT (DMMR) MEASTATIC COLORECTAL CANCER AFTER PRIOR FUOROPYRIMIDINE-BASED THERAPY IN COMBINATION WITH OXAPLATIN OR IRINOTECAN |
| 1. trastuzumab Deruxtecan | 4/15/2021 | n/a | treatment of adult patients with unresectable or metastatic HER2-positive breast cancer who have received prior treatment with trastuzumab emtansine (T-DM1) |
| 1. idecabtagene vicleucel | 5/26/2021 | n/a | adult patients with multiple myeloma who have received at least three prior therapies, including an immunomodulatory agent, a proteasome inhibitor, and an anti-CD38 antibody and who are refractory to their last treatment |
| 1. tepotinib | 5/27/2021 | n/a | adult patients with locally advanced or metastatic non-small cell lung cancer harbouring mesenchymal-epithelial transition tyrosine kinase receptor exon 14 skipping alterations |
| 1. selpercatinib | 6/15/2021 | n/a | monotherapy for the treatment of metastatic RET fusion-positive non-small cell lung cancer (NSCLC) in adult patients, RET-mutant medullary thyroid cancer (MTC) in adult and pediatric patients 12 years of age and older with unresectable advanced or metastatic disease, RET fusion-positive differentiated thyroid carcinoma in adult patients with advanced or metastatic disease (not amenable to surgery or radioactive iodine therapy) following prior treatment with sorafenib and/or lenvatinib |
| 1. pralsetinib | 6/30/2021 | n/a | the treatment of adult patients with rearranged during transfection (RET) fusion-positive locally advanced unresectable or metastatic non-small cell lung cancer (NSCLC) |
| 1. tafasatimab | 8/24/2021 | n/a | in combination with lenalidomide for the treatment of adult patients with relapsed or refractory diffuse large B-cell lymphoma (DLBCL) not otherwise specified, including BLBCL arising from low grade lymphoma, who are not eligible for ASCT |
| 1. sotorasib | 9/10/2021 | n/a | treatment of adult patients with Kirsten rat sarcoma viral oncogene homolog (KRAS) G12C-mutated locally advanced (not amenable to curative therapy) or metastatic non-small cell lung cancer (NSCLC) who have received at least one prior systemic therapy |
| 1. pemigatinib | 9/17/2021 | n/a | treatment of adults with previously treated, unresectable locally advanced or metastatic cholangiocarcinoma with a fibroblast growth factor receptor 2 (FGFR2) fusion or other rearrangement |
| 1. infigratinib phosphate | 9/27/2021 | n/a | adults with previously treated, unresectable locally advanced or metastatic cholangiocarcinoma with a fibroblast growth factor receptor 2 fusion or other rearrangement |
| 1. lurbinectedin | 9/29/2021 | n/a | adults with Stage III or metastatic small cell lung cancer (SCLC) who have progressed on or after platinum-containing therapy |
| 1. ranibizumab | 11/19/2021 | n/a | preterm infants for the treatment of retinopathy of prematurity (ROP) with zone I [stage 1 with plus disease (1+), stage 2 with plus disease (2+), or stage 3 with or without plus disease (3 or 3+)], or zone II [stage 3 with plus disease (3+)] or aggressive posterior ROP (AP-ROP) disease |
| 1. dostarlimab | 12/23/2021 | n/a | monotherapy for the treatment of adult patients with mismatch repair deficient or microsatellite instability-high recurrent or advanced endometrial cancer that has progressed on or following prior treatment with a platinum containing regimen |
